# GSK137, a potent small-molecule BCL6 inhibitor with *in vivo* activity, suppresses antibody responses in mice

**DOI:** 10.1016/j.jbc.2021.100928

**Published:** 2021-07-15

**Authors:** Andrew C. Pearce, Mark J. Bamford, Ruth Barber, Angela Bridges, Maire A. Convery, Constantinos Demetriou, Sian Evans, Thomas Gobbetti, David J. Hirst, Duncan S. Holmes, Jonathan P. Hutchinson, Sandrine Jayne, Larissa Lezina, Michael T. McCabe, Cassie Messenger, Joanne Morley, Melissa C. Musso, Paul Scott-Stevens, Ana Sousa Manso, Jennifer Schofield, Tom Slocombe, Don Somers, Ann L. Walker, Anastasia Wyce, Xi-Ping Zhang, Simon D. Wagner

**Affiliations:** 1 GlaxoSmithKline, Medicines Research Centre, Stevenage, UK; 2 Leicester Drug Discovery and Diagnostics, University of Leicester, Leicester, UK; 3 Leicester Cancer Research Centre and Ernest and Helen Scott Haematological Research Unit, University of Leicester, Leicester, UK; 4 GlaxoSmithKline, Collegeville, Pennsylvania, USA

**Keywords:** lymphoma, autoimmunity, drug discovery, immunology, lymphocyte, BCL6, BCL6 inhibitor, antibody response, BCL6, B-cell lymphoma 6, BTB, BR-C, ttk, and bab, clPARP, cleaved poly(ADP-ribose) polymerase, CSB, cell-staining buffer, DLBCL, diffuse large B-cell lymphoma, FBS, fetal bovine serum, GC, germinal center, IL, interleukin, IgG, immunoglobulin G, IgM, immunoglobulin M, KLH, keyhole limpet hemocyanin, PK, pharmacokinetic, POZ, pox virus and zinc finger, SLE, systemic lupus erythematosus, SMRT, silencing mediator for retinoid or thyroid hormone receptor, TE, target engagement, Tfh, CD4+ T-cell subset follicular helper, TNP, trinitrophenol

## Abstract

B-cell lymphoma 6 (BCL6) is a zinc finger transcriptional repressor possessing a BTB–POZ (BR-C, ttk, and bab for BTB; pox virus and zinc finger for POZ) domain, which is required for homodimerization and association with corepressors. BCL6 has multiple roles in normal immunity, autoimmunity, and some types of lymphoma. Mice bearing disrupted BCL6 loci demonstrate suppressed high-affinity antibody responses to T-dependent antigens. The corepressor binding groove in the BTB–POZ domain is a potential target for small compound-mediated therapy. Several inhibitors targeting this binding groove have been described, but these compounds have limited or absent *in vivo* activity. Biophysical studies of a novel compound, GSK137, showed an *in vitro* pIC_50_ of 8 and a cellular pIC_50_ of 7.3 for blocking binding of a peptide derived from the corepressor silencing mediator for retinoid or thyroid hormone receptors to the BCL6 BTB–POZ domain. The compound has good solubility (128 μg/ml) and permeability (86 nM/s). GSK137 caused little change in cell viability or proliferation in four BCL6-expressing B-cell lymphoma lines, although there was modest dose-dependent accumulation of G1 phase cells. Pharmacokinetic studies in mice showed a profile compatible with achieving good levels of target engagement. GSK137, administered orally, suppressed immunoglobulin G responses and reduced numbers of germinal centers and germinal center B cells following immunization of mice with the hapten trinitrophenol. Overall, we report a novel small-molecule BCL6 inhibitor with *in vivo* activity that inhibits the T-dependent antigen immune response.

B-cell lymphoma 6 (BCL6) is a transcriptional repressor that accomplishes its effects through binding of specific DNA recognition sequences by the C-terminal zinc fingers (1, 2), whereas corepressors BCL6 co-repressor, nuclear receptor co-repressor 1, and silencing mediator for retinoid or thyroid hormone receptor (SMRT) bind to the N-terminal BTB–POZ (BR-C, ttk, and bab for BTB; pox virus and zinc finger for POZ) domain and, in turn, recruit histone deacetylases. The midregion of BCL6 is also responsible for functionally important transcriptional repression through repression domain 2 (3) and mediates the proteasomal degradation of BCL6 through a PEST domain (peptide region that is rich in proline [P], glutamic acid [E], serine [S], and threonine [T]), (4) whereas the zinc-finger domain has also been reported to mediate interaction with the Eight-Twenty One protein (5).

*BCL6* was discovered as a gene involved in reciprocal chromosomal translocations (6), often with the immunoglobulin heavy chain locus, in about 25% of diffuse large B-cell lymphoma (DLBCL). *BCL6* mRNA and protein is expressed without translocation in about half of the cases of DLBCL (7) and has been suggested to be a therapeutic target in this disease (8, 9). BCL6 is also expressed in a subgroup of T-cell lymphoma, angioimmunoblastic T-cell lymphoma, derived from CD4^+^ T-cell subset follicular helper (Tfh) cells (10), as well as other B-cell lymphomas (follicular lymphoma (11) and Burkitt lymphoma) and other malignancies including breast cancer (12, 13) and non-small cell lung cancer (14) and could potentially be a therapeutic target in these conditions.

BCL6 has essential roles in normal immunity and characterisation of mice bearing homozygous disruptions of the BCL6 locus showed that it is required for high affinity antibody production in the germinal center response (15, 16). BCL6 is expressed in germinal center B-cells (17) but not naive B-cells or plasma cells and also in the CD4^+^ T-cell subset follicular helper (Tfh) T-cells, which are required for B-cell proliferation and the production of high affinity antibodies (18). BCL6 expression in both B-cells (19) and Tfh cells (20) is, therefore, essential for normal germinal center function, but the domains of the protein have non-redundant functions in the two lineages (21) such that the BTB-POZ domain is essential for BCL6 function in B-cells while its other domains have essential roles in Tfh-cells. Systemic lupus erythematosus (SLE) is a rare autoimmune condition whose prevalence may be rising in the UK (22). Plasma cells expressing the autoantibodies are believed to be important contributors to disease in both mice (23) and humans (24). The pathogenic IgG anti-DNA antibodies show somatic hypermutation (25), which are acquired during a germinal center response. Consistent with increased germinal center responses SLE patients have increased numbers of germinal centers, class-switched memory B-cells (26) and Tfh cells (27). Since pathogenic autoantibodies are responsible for some manifestations of disease, depletion of B-cells by therapeutic antibodies or routes to perturb B-cell function are considered avenues to treatment (28) in some patients. The evidence of increased germinal center responses driving the production of pathogenic autoantibodies in SLE (26, 29, 30) makes suppression of BCL6 function an attractive potential target for the disease.

The potential usefulness of a BCL6 inhibitor for some types of malignant and autoimmune disease has led several groups to develop approaches to perturb the cellular function of BCL6 with the ultimate aim of producing novel therapeutic agents. Detailed characterisation of co-repressor/BCL6 BTB-POZ domain co-crystal structures (31, 32) revealed the co-repressor residues binding in the lateral grooves formed by the interface between the BCL6 BTB-POZ homodimers and prompted the notion that a peptide corresponding to these co-repressor residues might interfere with BTB-POZ domain function. This appeared to be the case and the peptide slowed growth of DLBCL cells *in vitro* and *in vivo* and suppressed normal germinal center formation (8, 33). Others have subsequently developed different peptides to block co-repressor binding (34, 35) but the functional importance of the peptide binding site in the lateral groove of the BCL6 BTB-POZ domain prompted work to explore the development of small molecule inhibitors. A number of small molecule inhibitors with a variety of chemical structures have now been described (9, 36, 37, 38, 39, 40, 41, 42) and some of these are able to suppress DLBCL cell line proliferation and survival (9, 36, 41, 42). A small number of these compounds show some *in vivo* efficacy in repressing normal mouse antibody responses (37) or growth of cell line xenografts (37, 38).

We performed a high throughput screen to identify molecules capable of inhibiting the binding of a peptide derived from the SMRT co-repressor to the BCL6 BTB-POZ domain and identified hits with affinities in the 5 to 20 μM range. One of these hits was developed to produce the novel tool compound GSK137. We carried out a pharmacokinetic analysis of GSK137 and an *in vivo* study demonstrated reduction in specific antibody titers in response to immunization with T-dependent antigens at drug levels consistent with robust engagement with the BCL6 BTB-POZ domain target.

## Results

### Profile of GSK137

**Figure 1:**
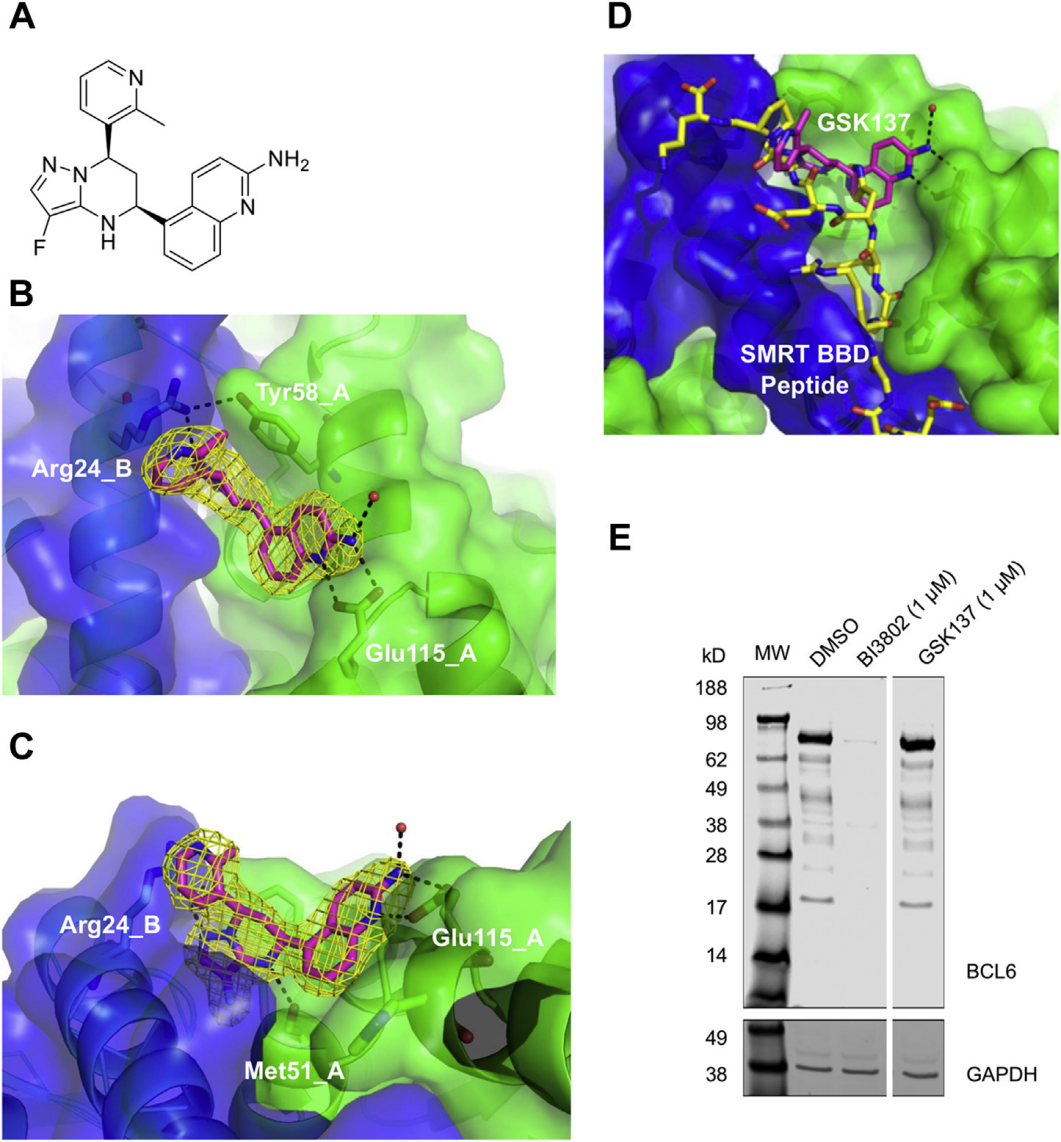
**Structure of GSK137 and binding to the BCL6 BTB–POZ domain.***A*, chemical structure of GSK137. *B*, X-ray crystal structure of GSK137 binding to the BCL6 BTB–POZ domain. One chain of the BTB–POZ homodimer is *green* (monomer A) and the other *blue* (monomer B). The *F*o–*F*c omit map electron density (contoured 3σ) around GSK137 (*yellow*) is indicated and the major residues making interactions; Tyr58, Glu115, and His116 on monomer A and Arg24 on monomer B. *C*, the view shown in *B* has been orthogonally rotated to demonstrate the Met51 hydrogen bond on monomer A. *D*, an overlay of the BCL6 BTB–POZ domain/GSK137 complex with the BCL6 BTB–POZ domain/SMRT corepressor BCL6-binding domain (BBD) peptide (*yellow*) complex (Protein Data Bank ID: 1R2B) showing the location of the ligand-binding sites at the lateral groove of the dimer interface and overlap with GSK137 binding. Monomers A and B are colored as before. *E*, Western blot of lysates from Farage DLBCL cells probed with an anti-BCL6 antibody (lane labeled DMSO). Other lanes show Farage lysates treated with the degrader compound BI3802 (1 μM for 24 h) and GSK137 (1 μM for 24 h). Molecular weight markers are indicated to the *left*. GAPDH is a loading control. The original uncut image is presented for transparency (Fig. S4). BCL6, B-cell lymphoma 6; BTB, BR-C, ttk, and bab; DLBCL, diffuse large B-cell lymphoma; DMSO, dimethyl sulfoxide; POZ, pox virus and zinc finger; SMRT, silencing mediator for retinoid or thyroid hormone receptor.

To identify chemical starting points for inhibiting BCL6 consideration was given to the fact that the desired mechanism of action was specifically to antagonise the binding of BCL6 to its BTB-POZ domain binding co-repressors, preserving function of other parts of the protein. Further, given the targeted interaction was a protein-protein interaction rather than a classical catalytic pocket it was decided to screen the full diversity of the GSK compound collection (∼1.7 million compounds) in a cell-free assay (time resolved fluorescence resonance energy transfer, TR-FRET) to maximise the chance of identifying binders. Inhibition of binding of a peptide derived from the SMRT co-repressor to the BTB-POZ domain of BCL6, by small molecules from the compound library, was determined. Groups of hit compounds from the high throughput screen were refined by further binding assays including surface plasmon resonance and nuclear magnetic resonance to derive a hit compound, which was the starting point for the development of GSK137 (Fig. 1A), a compound with a pyrazolo-tetrahydropyrimidine core substituted by aminoquinoline and methylpyridine moieties. The synthetic route is presented in Figure S3. GSK137 displaced peptides derived from the human SMRT co-repressor from the BCL6 BTB-POZ domain in a TR-FRET assay with a pIC_50_ of 8 (Table 1). It also displaced peptides from human nuclear receptor co-repressor 1 and BCL6 co-repressor corepressors, which bind in the same position as SMRT (32), both with a pIC_50_ of 7.6. We assessed activity of GSK137 against mouse and rat BCL6 and SMRT and determined the pIC_50_ to be 7.8 and 7.6, respectively. The ability of GSK137 to inhibit binding of intracellular full-length human BCL6 to BCL6-binding domain of SMRT (residues 1292–1500) in live cells was measured by NanoBret assay, and pIC_50_ was determined to be 7.3 (Table 1). BI3802 activity was consistent with the previously reported potency in cell-free and cellular assays (41).

**Table 1:** Key properties of GSK137

pIC_50_				Molecular weight (kD)	CAD solubility (μg/ml)	Chrom LogD/PFI	HSA binding (%)	Papp (nM/s)
Human			Mouse/rat					
TR-FRET		NanoBret cell	TR-FRET					
SMRT	nuclear receptor co-repressor 1/BCL6 co-repressor		SMRT					
8	7.6/7.6	7.3	7.8/7.6	374	128	2.3/6.3	91	86

pIC_50_ of the ability of the small molecule to inhibit binding of corepressor peptides from SMRT, nuclear receptor co-repressor 1, or BCL6 co-repressor and was measured by time-resolved FRET *in vitro*.

Cellular pIC_50_ was determined by NanoBret assay comprising Nano Luciferase fused at the N terminus of full-length human BCL6 and Halo tag fused at the C terminus of the BCL6-binding domain (residues 1292–1500) of human SMRT corepressor protein. Solubility was determined by a GSK in-house kinetic solubility assay with the amount of solubilized material being measured by charged aerosol detector (CAD). Effective hydrophobicity (log D) was determined chromatographically with the measurement modified to account for the number of aromatic rings (PFI, property forecast index). Percentage binding to human serum albumin (HSA) and permeability (Papp) were also determined.

The crystal structure of GSK137, in complex with the BCL6 BTB–POZ domain (residues 5–129) bearing changes Cys8Gln, Cys67Arg, and Cys84Asn to improve solubility of the protein (31), was obtained in order to explore the structure–activity relationship of BCL6 binding (Fig. 1, *B* and *C*). The 2.04 Å resolution crystal structure was generated from an apo BCL6 BTB–POZ domain crystal soaked with the compound. There was clear electron density for the ligand allowing the binding mode and absolute stereochemistry ([R]-chiral center at C6 and [S] at C8) to be determined. The compound binds in the lateral grooves in an equivalent manner on both sides of the interface utilizing the same key interactions with both protein chains of the BTB–POZ domain homodimer. Key interactions are observed between the three principal moieties of the compound and the homodimeric protein at both binding sites: (i) The aminoquinoline moiety is partially surface exposed but makes dual hydrogen bonds to the side chain of Glu115 residue and packs against a miniloop from Cys53 to Gly55, between two small helices of one monomer (monomer A in Fig. 1, *B* and *C*). (ii) The pyrazolo-tetrahydropyrimidine core lies deeper in the binding pocket, pi stacking against the aromatic side chain of Tyr58 and hydrogen bonding to Met51 main chain carbonyl, of monomer A, and Arg24 side chain of monomer B (Fig. 1*C*). Arg24 also hydrogen bonds to the Tyr58 hydroxyl (of monomer A [Fig. 1*B*]) to further stabilize the complex. (iii) The methylpyridine moiety is also solvent exposed like the aminoquinoline but is clearly stabilized through pi stacking against the Arg24 guanidinium group. An overlay of the BCL6 BTB domain–GSK137 complex with the BCL6 BTB domain–SMRT corepressor BCL6-binding domain peptide complex (43) (Fig. 1*D*) shows that GSK137 binds at the same lateral groove as the BCL6-binding domain of a known BCL6 corepressor and demonstrates the mechanism by which the compound could block corepressor binding.

Others have demonstrated that certain compounds binding to the BCL6 BTB–POZ domain are able to degrade intracellular BCL6 protein (41). One of these compounds, BI3802, as expected degraded BCL6 in Farage DLBCL cell line as determined by Western blot (Fig. 1*E*), but GSK137 did not show this activity.

Overall, GSK137 is a novel BCL6 BTB–POZ domain binder that antagonizes the binding of corepressors to BCL6 in cell-free and whole cell assays without causing loss of BCL6 protein.

### Effects on B-cell cancer cell lines

**Figure 2:**
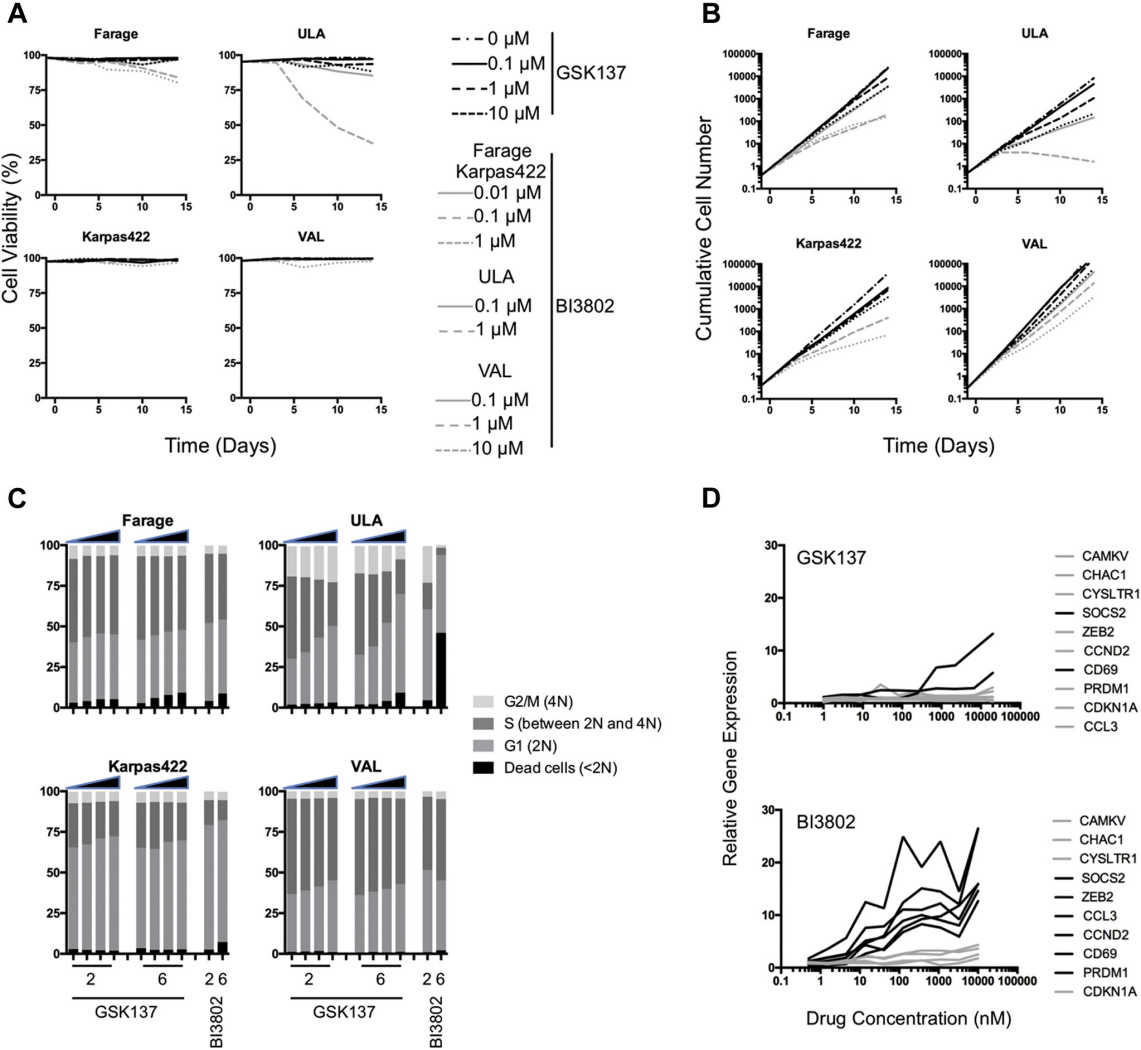
**Viability and proliferation of BCL cell lines in response to GSK137.***A*, cell viability of B-cell–expressing BCL cell lines Farage, Karpas422, ULA (all DLBCL), and VAL (BCL-6 translocated high-grade BCL) cell lines determined over 14 days and expressed as a percentage of the total cell population. GSK137 results are shown as *black lines*, and BI3802 are shown as *gray lines*. *B*, cumulative cell number was determined over 14 days. GSK137 results are shown as *black lines*, and BI3802 are shown as *gray lines*. For both (*A*) cell viability and (*B*) cumulative cell number, GSK137 concentrations used were 0 μM (*dot* and *dash line*), 0.1 μM (*solid line*), 1 μM (*dashed line*), and 10 μM (*dotted line*). BI3802 was used at 0.01 μM (*solid line*), 0.1 μM (*dashed line*), and 1 μM (*dotted line*) for Farage and Karpas422. For ULA, BI3802 was used at 0.1 μM (*solid line*) and 1 μM (*dashed line*). For VAL, BI3802 was used at 0.1 μM (*solid line*), 1 μM (*dashed line*), and 10 μM (*dotted line*). *C*, cell cycle analysis. Percentages of single cells in each of the four indicated populations (based on DNA content: <2 N [dead cells]; 2 N [G1]; between 2 N and 4 N [S phase]; and 4 N [G2/M]) are plotted for the cell lines Farage, Karpas422, VAL, and ULA. Increasing concentrations of GSK137 (0, 0.1, 1, and 10 μM) are indicated by the *black triangles* above the plots. BI3802 was used at one concentration (1 μM). Data were collected after 2 days or 6 days in culture. *D*, semiquantitative real-time PCR of a panel of ten genes (indicated to the *right*) in Farage cells. Expression values relative to DMSO are plotted against concentration of GSK137 (n = 1) or BI3802 (n = 2) following 72-h drug treatment. Genes whose expression was derepressed by drug treatment are indicated by *black lines*. BCL-6, B-cell lymphoma 6; DLBCL, diffuse large B-cell lymphoma; DMSO, dimethyl sulfoxide.

It was suggested that proliferation and survival of DLBCL cell lines expressing BCL6 are suppressed by small-molecule and peptide inhibitors of BCL6 (8, 9), but subsequent results have been mixed with some classes of inhibitor potently repressing DLBCL cell lines (37, 41, 42), whereas others show little effect (38, 39, 40). We selected three BCL6-expressing DLBCL model cell lines (Farage, Karpas422, and ULA) as well as a B-ALL model (VAL) to investigate the effects of GSK137. Of these cell lines, ULA and VAL bear a BCL6 translocation. Using cell counts and exclusion of cell-impermeant dye as a measure of viability, we found no major change in cell viability in the four cell lines (Fig. 2*A*) using a range of GSK137 concentrations from 0.1 to 10 μM. The degrader compound, BI3802, was used as a comparator molecule, and it only appreciably reduced viability in ULA at the highest concentration used (1 μM) from 6 days of culture onward. BI3802 suppressed increase in cell number to different degrees in the four cell lines with ULA being the most sensitive although Farage, Karpas422, and VAL showed some response (Fig. 2*B*). However, GSK137 demonstrated only minimal reduction in viability of ULA with no detectable change in viability or cumulative cell number in the other three cell lines.

Next, we determined cell cycle changes (Fig. 2*C*). ULA was again the most sensitive to BI3802 showing an increased fraction of cells in G1 at day 2 and ∼40% dead or dying cells at day 6 as compared with cells at the same time points that had not been treated with drugs. However, while GSK137 produced a modest dose and time-dependent G1 cell cycle arrest in ULA, there was no increase in dead or dying cells. In the other three cell lines (Farage, Karpas422, and VAL), both BI3802 and GSK137 caused only minor increase in the fraction of cells in G1. Collectively, both BI3802 and GSK137 had little effect on cell cycle or numbers of dead or dying cells in Farage, Karpas422, and VAL, but both compounds produced some accumulation of ULA cells in G1 with BI3802 having a greater effect and inducing some cell death at 6 days.

We carried out a limited investigation of gene expression changes in Farage cells following treatment with GSK137 or BI3802 by semiquantitative real-time PCR of a panel of 10 genes known, from the literature to be BCL6 target genes (44) (Fig. 2*D*). The degrader BI3802 caused dose-dependent changes to six of the ten genes, whereas GSK137 caused clear increase only in *SOCS2* with some increase in *CD69*.

Overall, GSK137 had no effect on cell viability, and only minor effects on cell growth or cell cycle arrest in the cell lines was tested, whereas a degrader compound, BI3802, had slightly more pronounced effects. Together, these results suggest only subtle effects of BCL6 inhibition in our panel of B-cell lymphoma cell lines.

### PK profile of GSK137 suggests suitability for *in vivo* studies

**Figure 3:**
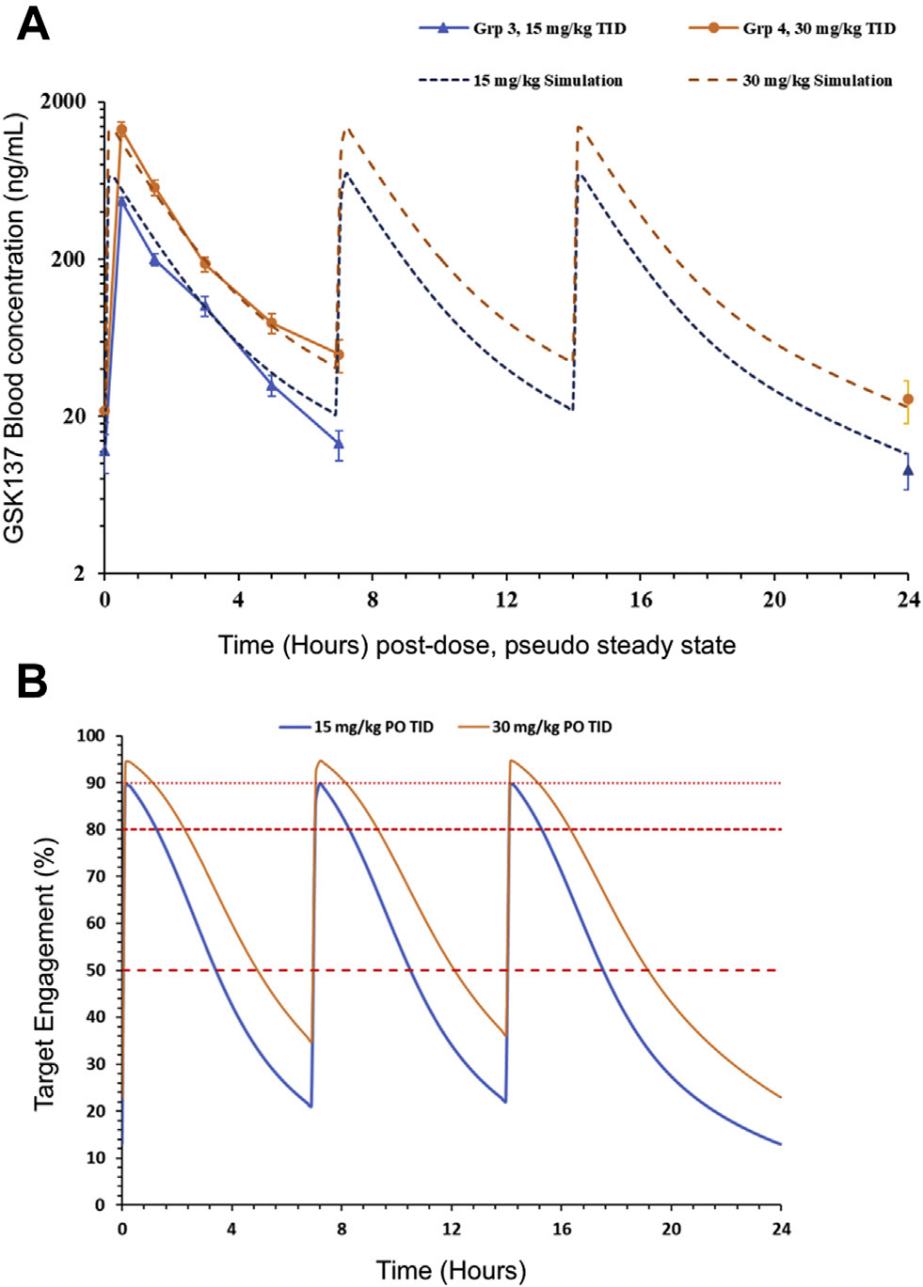
**Pharmacokinetics and target engagement (TE) following oral dosing of mice with GSK137.***A*, measured pharmacokinetic profiles of GSK137 (*solid lines*) and simulated steady pharmacokinetic profiles (*dashed lines*) at steady state following repeat oral, three times daily administration at doses of either 15 mg/kg (*blue lines*) or 30 mg/kg (*orange lines*). *B*, predicted percentage TE plot for GSK137 at simulated steady-state pharmacokinetic profiles following either repeat oral, three times daily administration at doses of either 15 mg/kg (*blue lines*) or 30 mg/kg (*orange lines*) based on cellular potency and blood-free drug levels. *Horizontal lines* indicate TE levels of 50, 80, or 90%.

Evidence from mice bearing constitutive homozygous disruptions of the *BCL6* locus indicates that BCL6 is required by B cells for high-affinity antibody responses, including implicating the BTB–POZ domain in this response (15, 16). A potent and cell-permeable BCL6 inhibitor with appropriate PK properties could, therefore, have clinical utility in suppressing antibody responses in autoimmunity. In order to assess whether acute blockade of BTB–POZ domain with a small-molecule drug was able to block high-affinity antibody responses, we investigated the ability of GSK137 to block the appearance of anti-trinitrophenol (TNP)–specific IgG following immunization with TNP–keyhole limpet hemocyanin (TNP–KLH) over 12 days. Doses for the study were selected based on prior pilot PK analysis, and the doses were required to give robust target engagement (TE). TE was modeled based on equating the level of free drug in blood to the cellular pIC_50_ for disrupting the BCL6–corepressor interaction in the NanoBret assay (Table 1). To confirm the actual exposures achieved in the study, concentrations of GSK137 in blood were measured at trough (before first daily dose of drug) on day 5 and day 12, and through the dosing period following the first daily dose on day 8 at 0.5, 1, 1.5, 3, 5, and 7 h, a composite “steady state” profile was obtained (Fig. 3*A*). The measured concentration–time profiles were fitted to a two-compartment PK model (*dotted lines* in Fig. 3*A*), and the concentration–time profile and simulated profiles described the measured concentration–time data well for both the 15 and 30 mg/kg GSK137 three times daily oral dosing. Mean trough concentrations ([day 5, predose]/[day 12 terminal]) and *C*_max_ (day 8, 0.5 h) increased 1.8- to 2.8-fold for a twofold increase in dose, with good dose separation. The simulated profiles were used to model TE with time and enable estimate of the time above the IC_50_, IC_80_, or IC_90_ for each daily dosing period (Table 2 and Fig. 3*B*). Following oral, three times daily dosing with 15 mg/kg, maximal TE was estimated to be 89.9% with an average TE area under the curve of 48%. The simulated profile suggested that GSK137 at 15 mg/kg did not achieve IC_90_ concentrations but was above the IC_50_ concentration for approximately 10 h of the daily dosing period. Simulated 30 mg/kg three times daily profiles were above the IC_90_ for at least 3 h, above the IC_50_ for approximately 15 h, with maximal TE of 94.7% and an average TE area under the curve of 62%. These considerations suggested that it would be possible to achieve biologically meaningful target inhibition by oral, three times daily dosing for GSK137.

**Table 2:** Pharmacokinetic and TE data for GSK137 administered orally and three times daily (orally, TID) at two doses (15 and 30 mg/kg)

Orally, TID	*C*_max_ (ng/ml)^a^	*T*_max_ (h)^a^	AUC_SS_ (ng.h/ml)^b^	Max TE (%)^c^	Average TE AUC (%)^d^	Time (h) above IC_xx_ (50/80/90)
15 mg/kg	471 ± 226	0.5	3400	89.9	48	10.3/3.8/0
30 mg/kg	1340 ± 141	0.5	6810	94.7	62	15.1/6.9/3.3

a *C*_max_ and *T*_max_ measured at day 8.

b Daily steady-state area under the curve (AUC_SS_) calculated from simulated pharmacokinetic profiles (linear trapezoidal/linear interpolation method).

c Calculated from the simulated *C*_max_, unbound using an *E*_max_ model with a Hill slope of 1, *E*_max_ 100%, and pIC_50_ of 7.4.

d AUC of TE data (%.h) divided by 24.

### Suppression of antibody responses in a mouse immunization model

**Figure 4:**
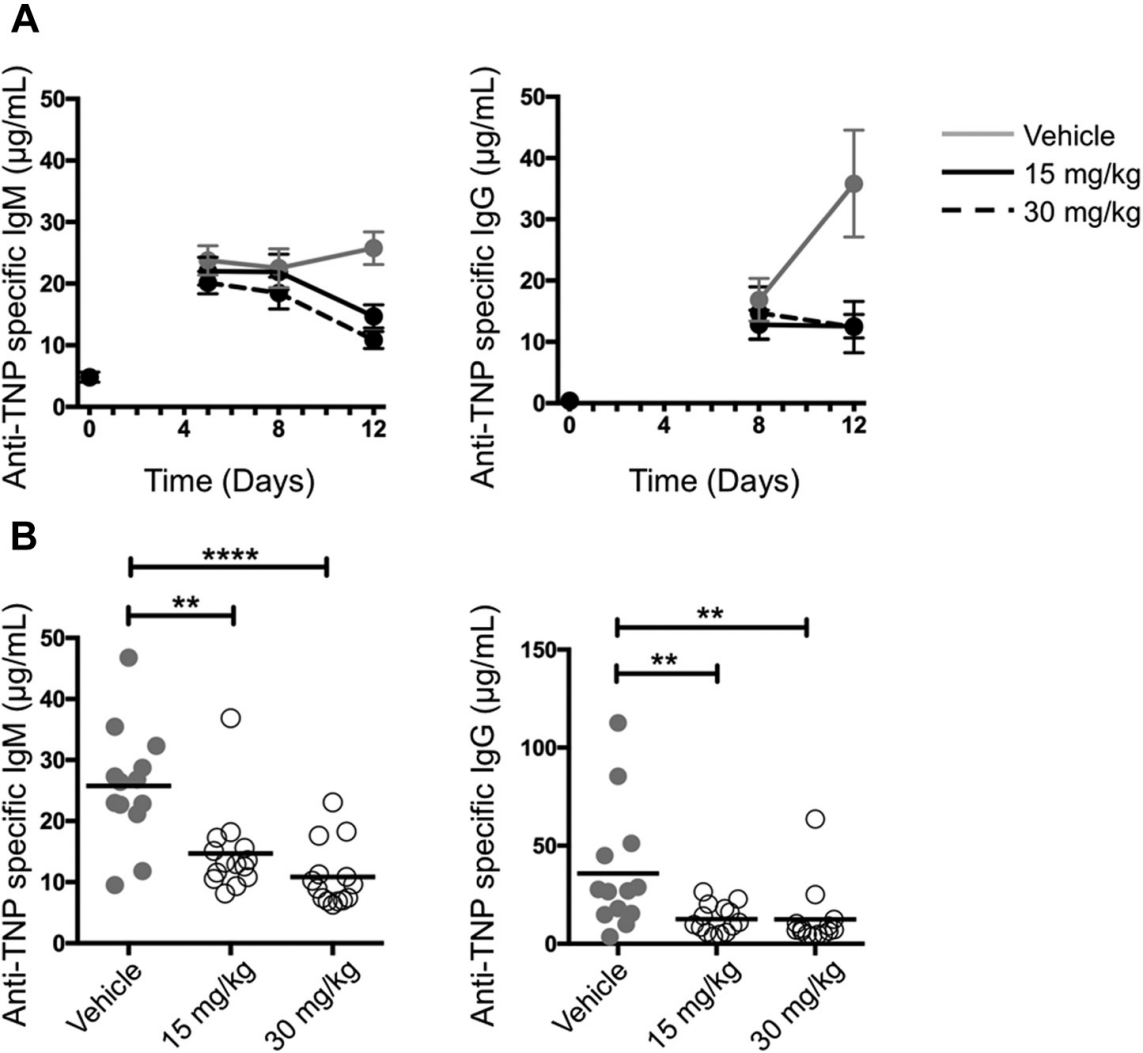
**Antibody responses.***A*, anti-TNP–specific IgM (*left-hand panel*) and immunoglobulin G (IgG) (*right-hand panel*) serum concentrations up to 12 days after immunization with TNP–KLH. Data are presented as mean ± SEM. Specific antibody levels in unimmunized animals are indicated at *T* = 0 (n = 4). Antibody levels in immunized but not drug-treated animals are indicated by the *gray line* (n = 13), immunized and treated with GSK137 (15 mg/kg) are indicated by *black solid line*, and immunized and treated with GSK137 (30 mg/kg) are indicated by *black dashed line*. *B*, scatter plot of antibody levels from individual mice at day 12 postimmunization in vehicle-and drug-treated groups. IgM responses (*left-hand panel*) and IgG responses (*right-hand panel*). The mean value is indicated by the horizontal bar. For the IgM responses, there are statistical differences between the vehicle-treated group and 15 mg/kg drug-treated group (Mann–Whitney *U* test, *p* = 0.0027) and vehicle-treated and 30 mg/kg group (*p* < 0.0001). For the IgG responses, there are statistical differences between the vehicle-treated and 15 mg/kg drug-treated group (*p* = 0.0056) and vehicle-treated and 30 mg/kg group (*p* = 0.0023). Statistical differences are shown as ∗∗*p* < 0.01 and ∗∗∗∗*p* < 0.0001. KLH, keyhole limpet hemocyanin; TNP, trinitrophenol.

To investigate whether the aforementioned dosing regimens and expected robust levels of TE by GSK137 could suppress GC-dependent antibody responses in mice immunized with the hapten, TNP, conjugated to KLH, the levels of anti-TNP antibodies were determined. Levels of serum TNP-specific immunoglobulin M (IgM) were elevated from day 5 in the vehicle-treated animals compared with naive animals. Treatment with GSK137 at 15 and 30 mg/kg did not inhibit TNP-specific IgM level on day 5 or day 8; however, statistically significant inhibition of IgM was seen by day 12 compared with vehicle (Fig. 4, *A* and *B*, *left-hand panels*) (Mann–Whitney *U* test; 15 mg/kg, *p* = 0.0027 and 30 mg/kg, *p* < 0.0001). As compared with vehicle-treated animals, mean IgM levels were reduced by 43% and 58% in mice treated with 15 and 30 mg/kg, respectively (Fig. 4*A*).

Levels of serum TNP-specific IgG detected in naive mice and mice treated with GSK137 or vehicle on day 5 were below the level of quantification (0.39 μg/ml) for most animals and are not shown. Levels of serum TNP-specific IgG were elevated on day 8 and further increased on day 12 in immunized and vehicle-treated mice (Fig. 4*A*, *right-hand panel*). Treatment with GSK137 at 15 and 30 mg/kg did not inhibit serum TNP-specific IgG levels on day 8, but a statistically significant repression was seen by day 12 as compared with vehicle treatment (15 mg/kg, *p* = 0.0056 and 30 mg/kg, *p* = 0.0023) (Fig. 4*B*, *right-hand panel*). As compared with vehicle-treated animals, GSK137 reduced mean IgG levels by 58% and 66% in mice treated with 15 and 30 mg/kg, respectively (Fig. 4*A*, *right-hand panel*). Therefore, GSK137 significantly repressed specific antibody responses in a mouse immunization model. Mouse weights did not vary significantly between vehicle- and GSK137-treated groups over the course of the experiment (Fig. S5) and, at the doses of GSK137 used, there were no overt signs of toxicity. Furthermore, levels of a panel of cytokines (interleukin-17 [IL-17], interferon gamma, IL-6, IL-10, tumor necrosis factor, IL-1β, IL-13, C-X-C motif chemokine ligand 1, monocyte chemoattractant protein 1, and IL-12) were either undetectable or, if detectable, did not differ significantly between vehicle- and GSK137-treated animals. Together, these results are consistent with specific targeted effects on BCL6-driven antibody responses.

### GC and B-cell and T-cell numbers

**Figure 5:**
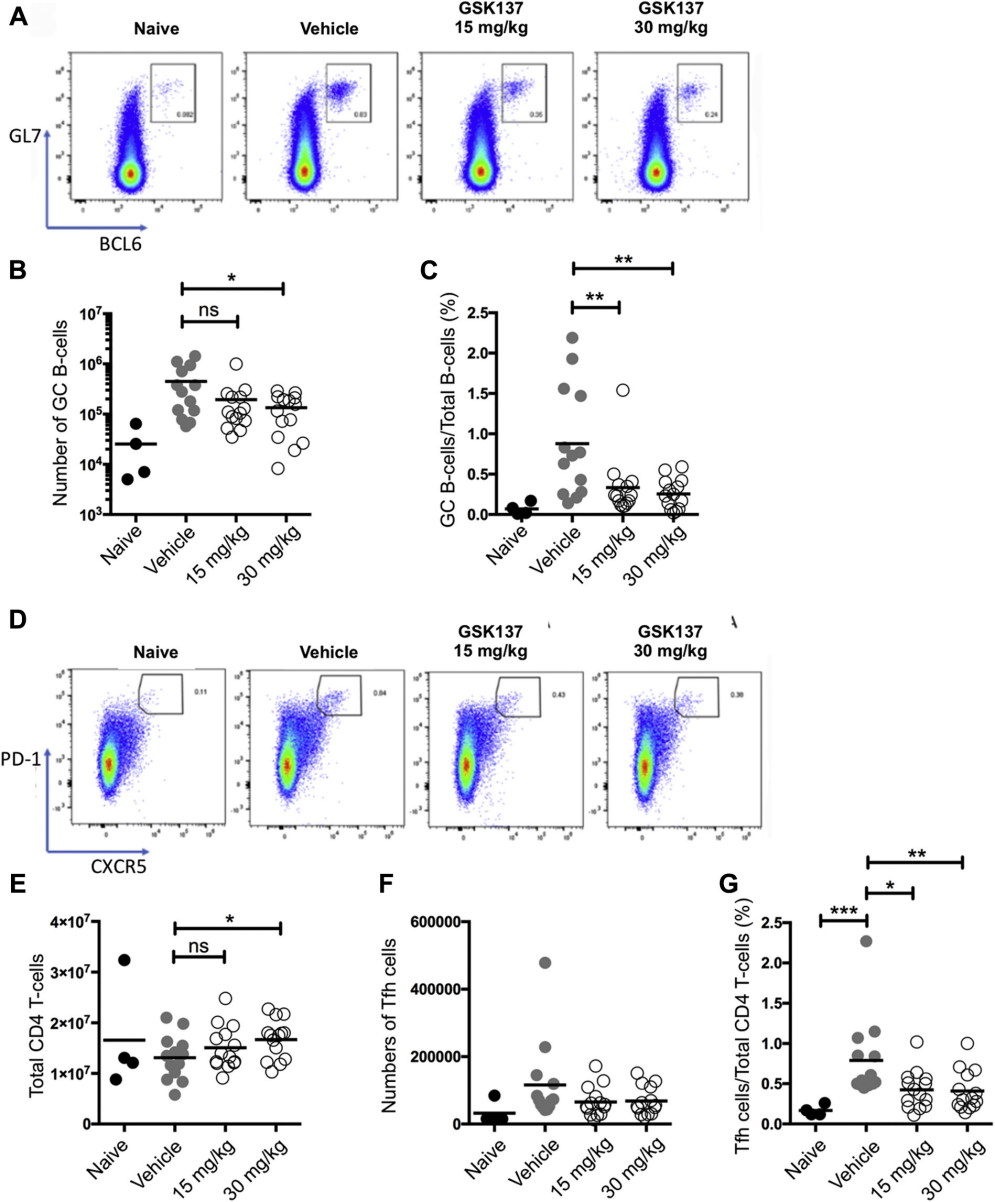
**Germinal center (GC) B-cell and Tfh cell numbers.***A*, representative plots gated on splenic CD19+ B cells showing frequencies of GC B cells identified by BCL6 and GL7 coexpression at day 12 after immunization with TNP–KLH. *B*, *C*, *E*, and, *F*, cell number in each of the four experimental groups at day 12 after immunization: naive unimmunized mice (n = 4), immunized and vehicle-treated (n = 13), immunized and GSK137 (15 mg/kg)-treated (n = 14), and immunized and GSK137 (30 mg/kg)-treated (n = 14) mice. Each point represents a single mouse, with bars indicating mean. *B*, absolute number of GC B cells in each of the four experimental groups. As compared with the vehicle-treated group, there is a statistically significant reduction in GC B cells with GSK137 (30 mg/kg) (Mann–Whitney *U* test, *p* = 0.04). *C*, splenic GC B cells expressed as a percentage of total CD19^+^ B cells in each of the four experimental groups. As compared with the vehicle-treated group, there are statistically significant reductions in the fraction of GC B cells with GSK137 (15 mg/kg) (Mann–Whitney *U* test, *p* = 0.0058) and GSK137 (30 mg/kg) (*p* = 0.0031). Horizontal bars indicate mean values. *D*, representative plots gated on splenic CD3e+ CD4+ T cells showing frequencies of T follicular helper cells identified by CXCR5 and PD-1 coexpression at day 12 after immunization with TNP–KLH. *E*, absolute number of total CD4^+^ T cells in each of the four experimental groups. As compared with the vehicle-treated group, there is a statistically significant increase in CD4^+^ T cells with GSK137 (30 mg/kg) (Mann–Whitney *U* test, *p* = 0.0032). *F*, numbers of T follicular helper cells per spleen in each of the four experimental groups. *G*, splenic T follicular helper cells expressed as a percentage of total CD4^+^ T cells. As compared with the vehicle-treated group, there is a statistically significant increase in the fraction of Tfh cells in the vehicle-treated group compared with the naive group (Mann–Whitney *U* test, *p* = 0.0008), and this is reduced with GSK137 (15 mg/kg) (*p* = 0.0144) and GSK137 (30 mg/kg) (*p* = 0.0041). Statistical differences are shown. ∗*p* < 0.05; ∗∗*p* < 0.01; and ∗∗∗*p* < 0.001. BCL6, B-cell lymphoma; KLH, keyhole limpet hemocyanin; ns, not significant; Tfh, CD4+ T-cell subset follicular helper cell; TNP, trinitrophenol.

At day 12 postimmunization, splenic GC B cells (detected as BCL6^+^GL7^+^ B cells [Fig. 5*A*]) and Tfh cells, detected as CXCR5^+^PD-1^+^ CD4^+^ T cells (Fig. 5*D*), in vehicle-treated mice were elevated compared with unimmunized animals (Fig. 5*B*). Treatment with GSK137 at 15 mg/kg reduced the number of GC B cells per spleen (mean numbers reduced by 53% compared with vehicle), and at a treatment dose of 30 mg/kg, the reduction achieved statistical significance (mean number of GC B cells reduced by 67%) (Mann–Whitney *U* test, *p* = 0.04) (Fig. 5*B*). The frequency of GC B cells within the total splenic B-cell compartment was evaluated, and GSK137, at both 15 and 30 mg/kg, significantly reduced mean numbers by 61% and 72%, respectively (Mann–Whitney *U* test: 15 mg/kg, *p* = 0.0058; 30 mg/kg, *p* = 0.0031) (Fig. 5*C*). Determination of plasmablasts and plasma cells in spleen (45) (CD19^+/int^CD138^+^) showed similar numbers in the GSK137-treated and vehicle-treated groups after immunization (Fig. S6), which we infer is due to low levels of terminal B-cell differentiation continuing to occur through a T-independent route not requiring BCL6 BTB–POZ domain function (46). Determination of the macrophage, dendritic cell, and natural killer cell markers (CD11c, CD11b, and CD161, respectively) also showed no difference between vehicle- and drug-treated groups (data not shown).

Comparing naive and vehicle groups demonstrates that immunization, with administration of vehicle alone, reduced mean total CD4^+^ T cells (Fig. 5*E*) and increased mean Tfh cells (Fig. 5*F*), but these effects did not reach statistical significance. GSK137 appeared to return mean CD4^+^ T-cell numbers to levels observed in naive mice, and this reached statistical significance at a dose of 30 mg/kg (Mann–Whitney *U* test, *p* = 0.032) but not at 15 mg/kg. As compared with animals receiving vehicle-alone treatment with GSK137 at 15 and 30 mg/kg reduced the mean absolute number of Tfh cells per spleen by 39% and 34%, respectively, compared with vehicle (Fig. 5*F*), but this was not statistically significant. The mean frequency of Tfh cells as a fraction of total splenic CD4^+^ T-cell compartment was significantly reduced by GSK137 at 15 and 30 mg/kg by 48% and 50%, respectively, compared with vehicle (Fig. 5*G*) (Mann–Whitney *U* test: 15 mg/kg, *p* = 0.014; 30 mg/kg, *p* = 0.0041). The data showed that GSK137 does not significantly alter the absolute numbers of Tfh cells, but the fraction of CD4^+^ T cells that are Tfh cells increased on immunization of nondrug-treated animals and was partially reduced toward normal levels by GSK137.

**Figure 6:**
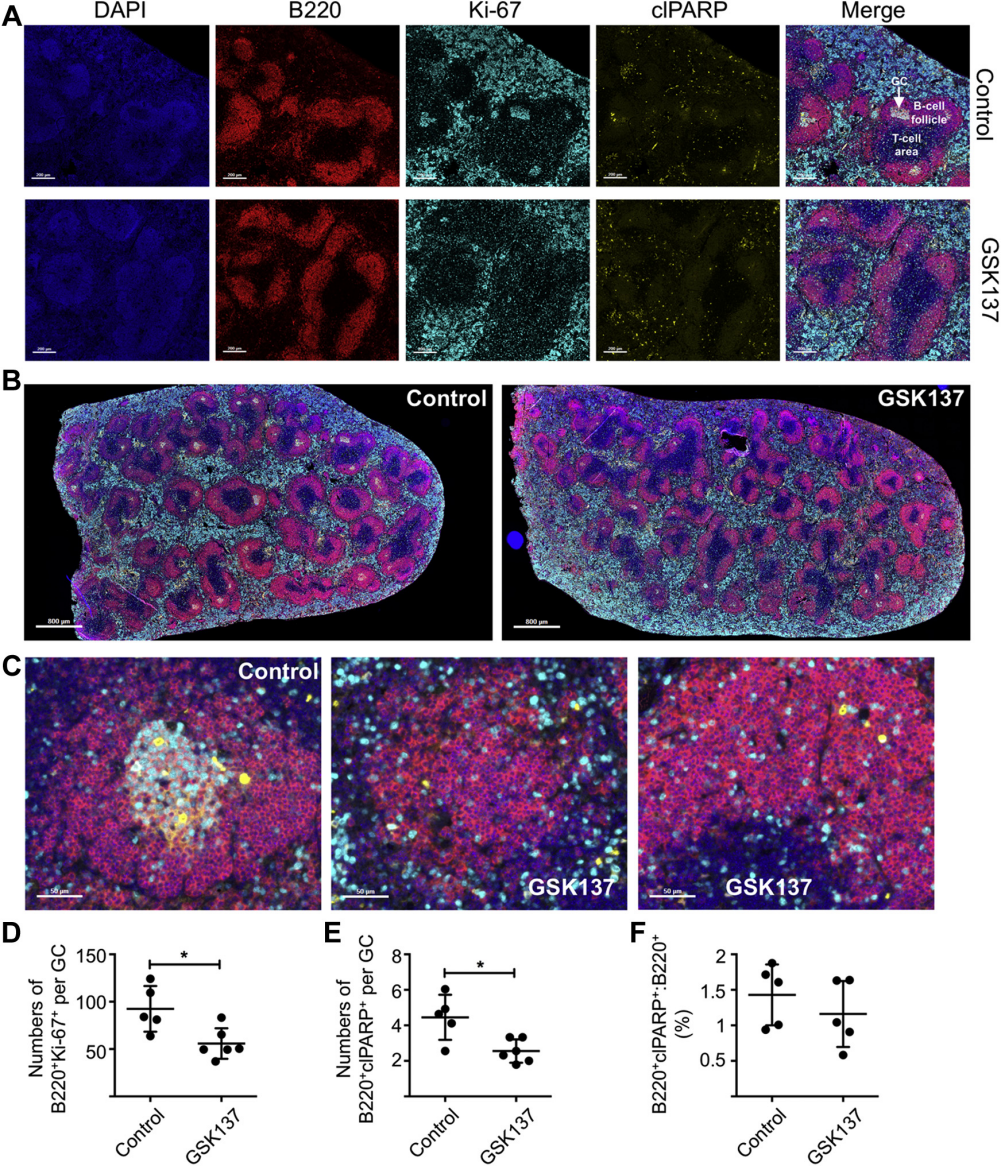
**Spleen immunohistochemistry from immunized and GSK137-treated mice.***A*, spleen sections from immunized mice, either untreated (control) or treated with GSK137, were stained with DAPI (*blue*), anti-B220 (*red*), anti-Ki-67 (*cyan*), and anti–cleaved PARP (*yellow*). A 200 μ size marker is indicated at *lower left*. The regions of the splenic *white pulp* are indicated: B-cell follicle, germinal center (GC), and T-cell area. *B*, low magnification images of spleen sections from immunized mice, which were untreated (control) or treated with GSK137, were stained as before. A 800 μ size marker is indicated at *lower left*. C, high magnification image of a GC from the spleen of a control group mouse showing cells staining with anti–cleaved PARP (*yellow*) and two images of B-cell follicles from immunized mice treated with GSK137. A 50 μ size marker is indicated at *lower left*. *D*, scatter diagram showing numbers of GCs per follicle in immunized and vehicle-treated animals *versus* immunized and GSK137 (15 mg/kg)-treated animals. Each data point is the mean of two technical replicates for each control animal (n = 6) or animal treated with GSK137 (n = 3). Mean and SD are indicated by the *horizontal line* and *error bars*. There is a significant difference between groups (Mann–Whitney *U* test, *p* = 0.04). *E*, scatter diagram showing mean fluorescence intensity of B220^+^Ki-67^+^, B220^+^clPARP^+^, and B220^+^ cells within manually identified GCs in control and GSK137-treated animals. Each point represents data from a spleen section of a separate mouse. Vehicle-treated group, n = 5 and GSK137-treated group, n = 6. Mean and SD are indicated by the *horizontal line* and *error bars*. There are significant differences between the two groups for B220^+^Ki-67^+^ cells (*p* = 0.044) and B220^+^ cells (*p* = 0.0087). Statistical differences are shown as ∗*p* < 0.05 and ∗∗*p* < 0.01. DAPI, 4′,6-diamidino-2-phenylindole; PARP, poly(ADP-ribose) polymerase.

GC formation is BCL6 dependent (15, 16). We, therefore, determined GC number by quantitative immunohistochemistry (clusters of B220 and Ki-67 expressing cells) in immunized plus vehicle (control) and immunized plus GSK137 (15 mg/kg) animals (Fig. 6, *A* and *B*). The number of GCs per follicle was reduced from 0.5 ± 0.11 (mean ± SD) in the vehicle-treated group to 0.27 ± 0.06 in the GSK137-treated group (Mann–Whitney *U* test, *p* = 0.04) (Fig. S7). There were also significant reductions in numbers of B220^+^Ki-67^+^ cells within each GC (unpaired *t* test, *p* = 0.01) (Fig. 6*D*). Apoptosis is a feature of normal GCs, and as expected, cells expressing cleaved poly(ADP-ribose) polymerase (clPARP) were readily detected within GCs in the spleens of vehicle-treated mice (Fig. 6*C*) with scattered apoptotic cells within the B-cell follicles in both control and GSK137-treated groups. In line with an overall reduction in GC size in the spleens of drug-treated animals, there was a significant reduction in numbers of B220^+^clPARP^+^ cells per GC (unpaired *t* test, *p* = 0.01) (Fig. 6*E*). There was no significant difference in the fraction of B220^+^ cells expressing clPARP within the splenic B-cell follicles between mice treated with GSK137 and control animals (Fig. 6*F*) showing that the novel agent does not increase apoptosis through nonspecific effects *in vivo*.

## Discussion

We produced GSK137, a novel, potent, and cell permeable small-molecule inhibitor of corepressor binding to the BCL6 BTB–POZ domain. This molecule did not have degrader activity and had little activity in reducing viability or suppressing growth of B-cell lymphoma cell lines that expressed BCL6. Its PK profile suggested that GSK137 might achieve exposures sufficient to deliver robust levels of TE and to have *in vivo* activity. Accordingly, we investigated its effects in suppressing specific antibody formation in immunized mice.

GSK137 reduced GC number in response to immunization and caused mild repression of IgM and pronounced repression of IgG levels, which is consistent with the pattern of immune response to hapten reported in mice bearing homozygous deletions of the BCL6 locus (15, 16). Circulating IgM^+^ human B cells can express CD27, a marker characteristic of memory B cells (47). These cells have subsequently been shown to bear somatically hypermutated antibody genes (48) and are considered to be derived from a GC response (49). Reduction in specific IgM titers by GSK137 is, therefore, likely to be due to an on-target effect suppressing BCL6 BTB–POZ domain function in GC B cells.

Although BCL6 is required for both GC B cells and T cells, the protein complexes of which it is a part may be lineage specific. Knock-in mutation to abolish BCL6 BTB–POZ domain function suppressed differentiation of GC B cells and antibody responses (21) but did not affect Tfh development. Genetic ablation of the BCL6 BTB–POZ domain, however, caused a reduction in Tfh cells as a fraction of CD4^+^ T cells, which was assumed to be secondary to lack of GC B cells (21). GSK137 reduced numbers of GC B cells and GCs, as we showed by flow cytometry and immunohistochemistry, respectively (Figs. 5 and 6), and it also caused significant reductions in the fraction of Tfh cells in the CD4^+^ T-cell pool, similar to the observations made in mice bearing mutations of the BCL6 BTB–POZ domain and consistent with GSK137 having *in vivo* on-target effects.

Furthermore, our results demonstrate that acute intervention with a small-molecule blocker of the BTB–POZ domain is sufficient to inhibit T-dependent antigen responses, confirming that the effects previously reported in mice bearing constitutive BTB–POZ domain mutations are not because of reprogramming of the immune system during development. These results support the possibility that delivery of a small-molecule inhibitor may be beneficial in some antibody-driven pathologies, such as SLE, and this warrants further investigation in animal models. Speculatively, a BCL6 inhibitor whose mechanism of action is specifically to suppress BTB–POZ domain function while not affecting the other BCL6–protein interaction domains or overall protein expression may have fewer toxicities and a better therapeutic index than other molecules, and this is supported by animal studies: mice bearing BCL6 BTB–POZ domain mutations show defective GC function (21) but do not show the severe inflammatory phenotype of mice bearing disruptions of the BCL6 locus (15, 16).

BCL6 is linked to the development of some cases of DLBCL through recurring genetic alterations that include mutations increasing BCL6 expression (50, 51, 52, 53) and a reciprocal translocation involving BCL6 observed in ∼25%. In addition, patients with DLBCL with higher levels of BCL6 mRNA and protein levels show a better prognosis as compared with those with lower levels (7) supporting the role of BCL6 in the biology of subtypes of DLBCL and suggesting that BCL6 expression is associated with higher response rates to current chemoimmunotherapy. However, it is not clear whether BCL6 is required for maintenance, as opposed to development of human DLBCL, and indeed a mouse model shows that transient BCL6 expression at an early stage of development is sufficient to drive development of lymphomas (54). Overall, the data have been interpreted to suggest that DLBCL may be amenable to treatment by BCL6 inhibitors.

GSK137 with an affinity for the BCL6 BTB–POZ domain of 10 nM (pIC_50_ = 8; Table 1) has a potency of the same order as BI3802 (<3 nM) but produced virtually no detectable reduction in proliferation, only minor derepression of BCL6 target genes and modest G1 cell cycle increases in lymphoma cell lines including DLBCL cell lines. These findings are in line with others who have shown that nondegrader BCL6 inhibitors have more restricted effects on suppression of proliferation of DLBCL cell lines than degrader molecules (41). Similarly, the BCL6 inhibitors 79-6 (9) and its subsequent development, FX-1 (36) have antiproliferative effects on several DLBCL cell lines, whereas others have found that small-molecule BCL6 inhibitors have only weak antiproliferative activity across panels of cell lines (39). Again, some BCL6 inhibitors have been reported to cause apoptosis (9), whereas others have shown that potent BCL6 degrader molecules do not cause apoptosis (41). CRISPR/Cas9-mediated ablation of BCL6 in DLBCL cell lines caused G1 cell cycle arrest but no apoptosis (55) suggesting that on-target BCL6 inhibition may indeed not cause cell line apoptosis. One possibility, consistent with the greater potency of degrader molecules, is that protein interactions mediated by domains of BCL6 other than the BTB–POZ domain are required for maintenance of full proliferation in DLBCL cell lines, but it is not clear why inhibition of BTB–POZ function alone has so little effect. It will be interesting in future work to investigate the effects of nondegrader BCL6 BTB–POZ domain inhibitors on DLBCL cells that are likely to be more representative of primary human tumors such as patient-derived xenograft models. Overall, the effects of GSK137 provide further information in an ongoing debate on the effects of inhibition of parts or all BCL6 function on DLBCL cell lines.

We report a thorough PK analysis of compound exposure and suppression of IgG antibody responses by an orally active BCL6 BTB–POZ domain inhibitor. Our work is encouraging in showing biologically and potentially clinically relevant *in vivo* effects of nondegrader BCL6 BTB–POZ domain inhibitor in a small-molecule inhibitor with drug-like properties compatible with oral dosing.

## Experimental procedures

### Crystallography

Purified BCL6 BTB domain (at 9.05 mg/ml in 20 mM Tris [pH 8.5], 250 mM NaCl, 5% glycerol, and 5 mM DTT) was crystallized at 20 °C by sitting drop vapor diffusion with well buffer (11% PEG 3350, 20% glycerol, 0.014 M Bis–Tris propane [pH 7.0], and 0.086 M Bis–Tris propane [pH 8.0]) and seed stock (in 25% PEG 3350, 20% glycerol, and 0.1 M Bis–Tris propane [pH 6.5]) at drop ratio of 1:0.8:0.2, respectively. An apocrystal was soaked in the same buffer as the seed stock and containing GSK137 (25 mM) and 10% dimethyl sulfoxide for 42 h at 20 °C. The soaked crystal was cryocooled in liquid nitrogen, and diffraction data were collected at 100 K using an FR-E+ Superbright X-ray generator/Saturn A200 CCD detector system (Rigaku). The data obtained from the crystal (Table S1) were processed using Global Phasing autoPROC (56) running XDS (57) and scaling with AIMLESS (58) within CCP4 (59) followed by Global Phasing STARANISO (60). The structure was solved by Fourier synthesis and refined using REFMAC (https://www.ccp4.ac.uk/html/refmac5.html) (61). Model building was carried out using COOT software (https://www2.mrc-lmb.cam.ac.uk/personal/pemsley/coot/) (62). The compound dictionary file used in refinement was created by GRADE (http://grade.globalphasing.org/cgi-bin/grade/server.cgi) (63). The final Rfactor (and Rfree) achieved for the structure was 18.0% (24.1%). Figures were created using PyMOL (Schrödinger, LLC) (64).

### B-cell lymphoma cell lines

Farage cells were cultured in RPMI1640 (1×) + 20% heat-inactivated fetal bovine serum (FBS), ULA in Iscove's modified Dulbecco's medium + 20% FBS, whereas Karpas422 and VAL were cultured in RPMI1640 + 20% heat-inactivated FBS, all with the addition of 1% Glutamax and 1% sodium pyruvate.

### Immunization model

Animal studies were reviewed and approved by the University of Leicester Animal Welfare and Ethical Review Body and GSK Animal Welfare and Ethical Review Body. All studies were carried out in accordance with the GSK Policy on the Care, Welfare and Treatment of Animals and with the Animals (Scientific Procedures) Act 1986.

Male CD1 mice (30–40 g, 8–9 weeks of age) were randomized into study treatment groups, housed individually and allowed to acclimatize under standard laboratory conditions for a minimum of 5 days prior to commencement of the study. Mice received food and water ad libitum. The experiment was performed in a certified dedicated *in vivo* experimental laboratory at the GlaxoSmithKline Medicines Research Centre, Stevenage, United Kingdom. The four treatment groups were (1) unimmunized and untreated (naive group) (n = 4); (2) intraperitoneal vehicle (n = 13), (3) GSK137 15 mg/kg (n = 14), and (4) GSK137 30 mg/kg (n = 14). Groups (2) to (4) were given intraperitoneal immunization with TNP–KLH (100 μg) on day 0, vehicle was 1% methylcellulose in water + 0.1% sodium lauryl sulphate, and compound or vehicle was given to groups (2) to (4) by oral gavage three times per day from day −1 to day +12. Blood samples were collected by tail vein for measurement of compound, IgM and IgG levels on days 0, 5, 8, and 12 ahead of immunization/first daily dose. On day 8, blood samples were also taken (n = 7) at 0.5, 1.5, 3, 5, or 7 h after first daily dose. On day 12, spleens were collected, weighed, and placed into Dulbecco's modified Eagle's medium on wet ice for analysis by flow cytometry.

Immunohistochemistry of GCs in spleens was obtained from a pilot experiment to test immunization conditions carried out at the University of Leicester using groups of C57BL/6J mice. One group of mice was immunized with TNP–KLH (100 μg) followed by treatment with GSK137 15 mg/kg by oral gavage three times per day from day −1 to day +12 (n = 3), whereas the other was treated with vehicle by oral gavage three times per day from day −1 to day +12 following immunization (n = 6).

### Spleen immunohistochemistry

Formalin-fixed paraffin-embedded sections of mouse spleen were stained with antimouse B220/CD45R (BD Biosciences; catalog no. 550286), anti-clPARP (Abcam; ab32064), and anti-Ki67 (Abcam; ab15580) antibodies using Opal Multiplex IHC detection kit (NEL810001KT; Akoya Biosciences). The sections were counterstained with 4′,6-diamidino-2-phenylindole for 5 min and mounted with ProLong Diamond Antifade mountant (Invitrogen; P36970). Images were obtained using Vectra Polaris multicolor fluorescence scanner (Akoya Biosciences). Quantification was carried out by inForm image analysis software (Akoya Biosciences). Normality tests were carried out for the B220^+^Ki67^+^ and B220^+^clPARP^+^ data. Both datasets were normally distributed, and accordingly, statistical significance was determined by unpaired *t* test.

### Measurement of antibodies and cell numbers in immunization model

For antibody measurements, TNP–bovine serum albumin–coated plates were blocked, and then, standards or serum samples diluted 1:1000 or 1:2500 were incubated for 2 h at room temperature before biotinylated antimouse IgG or IgM antibodies were allowed to bind for 1 h at room temperature and detected using streptavidin-bound horseradish peroxidase incubated for 30 min, followed by the addition of 3,3′,5,5′-tetramethylbenzidine substrate for 15 min. The reaction was stopped, and the plates read at 450 nm by spectrophotometry. Plates were washed between each incubation step to remove unbound reagent. Data analysis was carried out in GraphPad Prism 5 (GraphPad Software, Inc) software using a four-parameter logistic fit of the standards response.

For cell measurements, spleens were passed through a 40 μM cell strainer, red blood cells were lysed, and cells were resuspended in medium, counted, and 1 × 10^6^ cells plated for staining. Cells were washed in PBS and resuspended in 100 μl viability dye (diluted 1:1000 in PBS) for 15 min at room temperature. About 100 μl cell-staining buffer (CSB; BioLegend; catalog no. 420201) was added to each well before centrifugation and removal of supernatants, and cell pellets were resuspended in 100 μl relevant cocktail of antibodies (Table S2) for surface staining and incubated for 30 min at 4 °C. Cells were then washed in CSB and resuspended in 200 μl of FoxP3 fixation and permeabilization buffer for 30 min for staining of intracellular markers for 30 min. Cells were washed in PermWash buffer and resuspended in CSB (250 μl) before acquisition of a Cytek Aurora spectral cytometer (Cytek). Gating was carried out as shown (Figs. S1 and S2), and data were analyzed on FlowJo 10 (Becton Dickinson). Cell frequency was used to calculate an absolute cell number per tissue from the total live cell counts from whole homogenized spleen. Data were normalized against background GC and Tfh quantified in naive mice prior to performing percent inhibition calculations.

### PK sample processing and analysis

Mouse blood/water (50/50) samples were analyzed for GSK137 using an analytical method based on protein precipitation, followed by HPLC/MS–MS analysis. The lower limit of quantification for GSK137 was 1.2 ng/ml with a higher limit of quantification of 3000 ng/ml. A two-compartment PK model was used to describe the measured composite mean steady-state oral concentration–time profiles. A three times per day dose simulation was performed using the two-compartment PK model parameters generated to simulate the repeat dose concentration–time profile. All modelings were conducted in Phoenix64 (WinNonLin) version 8.1 (Pharsight).

## Data availability

All data are contained within the article apart from the X-ray crystallography data (dataset) 2020. Human BCL6 BTB domain is in complex with GSK137 (Protein Data Bank ID: 7BDE).

## Supporting information

This article contains supporting information.

## Conflict of interest

The authors declare that they have no conflicts of interest with the contents of this article.
