# Supplementary material for: GSK137, a potent small-molecule BCL6 inhibitor with in vivo activity, suppresses antibody responses in mice
Source: J Biol Chem. 2021 Jul 15;297(2):100928. doi: 10.1016/j.jbc.2021.100928 (PMC8350397; doi:10.1016/j.jbc.2021.100928)
Supplement: Supporting information [file mmc1.docx]

**Supporting Information**

A potent small molecule BCL6 inhibitor, GSK137, with *in vivo* activity suppresses antibody responses in mice.

**Andrew C. Pearce, Mark J. Bamford, Ruth Barber, Angela Bridges, Maire A. Convery, Constantinos Demetriou, Sian Evans^2^, Thomas Gobbetti, David J. Hirst, Duncan S. Holmes, Jonathan P. Hutchinson, Sandrine Jayne, Larissa Lezina, Michael T. McCabe, Cassie Messenger^1^, Joanne Morley, Melissa C. Musso, Paul Scott-Stevens, Ana Sousa Manso, Jennifer Schofield, Tom Slocombe, Don O. Somers, Ann L. Walker, Anastasia Wyce, Xi-Ping Zhang, Simon D. Wagner**

**Contents**

**Materials and Methods** Pages 2 - 11

**Table S1 -** Data collection and refinement statistics (Molecular replacement).

**Table S2 -** Antibody panels for flow cytometry.

**Table S3 -** Reagents and antibodies for flow cytometry.

**Table S4 -** TaqMan assays used in the study. All assays were purchased from Life Technologies.

**Figure S1 -** Sequential gating strategy for identification of germinal center B cells.

**Figure S2 -** Sequential gating strategy for identification of Tfh cells.

**Figure S3 -** General synthetic routes to GSK137.

**Figure S4 -** Western blot from Figure 1E shown complete for transparency purposes.

**Figure S5** - Change in mouse body weight over the course of the immunisation experiment.

**Figure S6** - Plasmablast numbers in vehicle and GSK137 treated groups.

**Figure S7** - Germinal center numbers in vehicle and GSK137 treated groups.

**Materials and Methods**

*Western blots*

Cell pellets were lysed in RIPA buffer containing protease/phosphatase inhibitor cocktail and benzonase, protein concentration determined and samples prepared in reducing conditions and 20 µg of protein loaded per lane. Proteins were then transferred onto a nitrocellulose membrane and probed with rabbit BCL6 antibody (Abcam, Cambridge, UK; ab33901) and mouse GAPDH antibody (Abcam; ab9484) at 1:2000 dilution overnight at 4°C. Blots were probed for 1 hour at room temperature with secondary antibodies (goat anti-rabbit LI-COR, 926-32211 and goat anti-mouse LI-COR, 926-68070) at 1:10,000 dilution). Images were obtained on a LI-COR Odyssey CLx Infrared Imaging System using Image Studio software (Version 5.2).

*Viability and cumulative cell number analysis*

Farage, ULA, Karpas422, and VAL cells were seeded into T25 tissue culture flasks in a total volume of 7 mL per flask at densities of 5 x10^4^, 7 x10^4^, 5 x10^4^, and 4 x10^4^ cells/mL, respectively and incubated overnight at 37 ˚C and 5% CO2. Cells were then treated with a 3-point titration of GSK137 (0.1 µM, 1 µM, and 10 µM) throughout a 14-day time course. Treatments with multiple concentrations of BI3802 were also included for comparison (Farage and Karpas422 cells were treated with 0.01 µM, 0.1 µM, and 1 µM BI3802; ULA cells were treated with 0.1 µM and 1 µM BI3802; VAL cells were treated with 0.1 µM, 1 µM, and 10 µM BI3802). Viability (trypan blue exlcusion) and cumulative cell number were measured on a Vi-CELL XR 2.03 (Beckman Coulter, Brea, CA, USA) on day of seeding and on days 3, 6, 10, and 14.

*Cell cycle analysis*

Farage, ULA, KARPAS-422, and VAL cells were seeded into 6-well tissue culture dishes in a total volume of 6 mL at densities of 6.25 x10^3^, 7.81 x10^3^, 6.25 x10^3^, and 1.56 x10^3^ cells/mL, respectively and incubated overnight at 37 ˚C and 5% CO_2_. Cells were then treated with 0.1 µM, 1 µM, or 10 µM GSK137 or 1 µM BI3802 for 2 or 6 days at 37 ˚C and 5% CO_2_. Cell cycle analysis was performed using the Click-iT EdU Alexa Fluor 647 Flow Cytometry Assay Kit (Thermo Fisher Scientific, Waltham, MA, USA) following the manufacturer’s instructions, with volumes adjusted for a 384-well U-bottom plate, and with an approximately 1 hour treatment with 10 μM EdU. After EdU treatment, approximately 2x10^5^ cells per well were transferred into a 96-well, non-tissue culture treated U-bottom plate and stained with 6.7μg/ml final FxCycle Violet Stain for 30 minutes incubation at room temperature. Cells were then analyzed on a flow cytometer (BD FACS Canto 10 or LSR FortessaTM X-20 (Becton Dickinson, Franklin Lakes, NJ, USA)) with compensation each day using one cell line and data analyzed using FlowJo software (version 10.3). Percentages of cells in each phase of the cell cycle were then averaged across technical replicates.

*BCL6 BTB domain protein expression for enzyme assays and crystallography*

Human, mouse and rat BCL6-Flag-6H-Avi (5-129) C8Q, C67R, C84N proteins were expressed in E. coli according to standard procedures and purified on Ni-NTA, followed by ion exchange on a Source 30Q column and size exclusion on an Superdex 200 column in a buffer of 20 mM Tris, 250 mM NaCl, 5 mM DTT, 5% glycerol, pH 8.5. All protein purities were estimated to be >90% by SDS-PAGE and the expected masses were confirmed by MS analysis. For TR-FRET assay protein was enzymatically biotinylated by treatment of protein at 100 μM with a 2 fold molar excess of biotin in the presence of 2 mM ATP and 1 μM BirA enzyme in a buffer of 50 mM bicine pH 8.3, 10 mM MgCl2 at 30 ˚C for 1 hour, followed by extensivedialysis against 50 mM Tris-Cl pH 8.0, 100 mM NaCl, 1 mM DTT using a 3.5 kDa cut-off membrane. Biotinylation was confirmed by MS analysis and concentration was determined by Bradford assay, after which protein was stored in aliquots at -80 ˚C.

For crystallogaphy studies, human BCL6-Flag (5-129) C8Q, C67R, C84N was expressed in E. coli according to standard procedures and purified on anti-FLAG agarose beads followed by size exclusion on a Superdex 200 column in a buffer of 20 mM Tris, 250 mM NaCl, 5 mM DTT, 5% glycerol, pH 8.5. Protein purity was estimated to be >90% by SDS-PAGE and the expected mass was confirmed by MS analysis.

*Peptides and other biochemical assay reagents*

Acceptor-labelled SMRT peptide H_2_N-LVATVKEAGRSIHEIPRC(D2)-OH was dissolved in water by mass to give a 500 μM stock solution which was stored at -20 ˚C. Acceptor-labelled NCOR peptide H_2_N-ITTIKEMGRSIHEIPRC(Alexa Fluor 647)-OH and acceptor-labelled BCOR peptide H_2_N-RSEIISTAPSSWVVPGPC(Alexa Fluor 647)-OH were dissolved to a nominal concentration of 1 mM in DMSO, and stock solution concentration was determined by absorbance at 650 nm using an extinction coefficient of 270,000 M-1cm^-1^. Lance Europium W1024 labelled Streptavadin was obtained from Perkin Elmer (catalogue number AD0062). Alexa fluor 647 labelled anti-GST was obtained from Thermo Fisher (catalogue number MA4-004-A647).

*NanoBRET assay reagents*

Plasmids encoding Nano Luciferase fused at the N-terminus of full-length human BCL6 and encoding Halo tag fused at the C-terminus of the BCL6 binding domain (1292-1500) of human SMRT co-repressor were generated under contract by Promega Corporation. HEK293-WT cells were from ATCC (CRL-1573). Fugene HD transfection reagent (catalogue number E2311), HaloTag NanoBRET 618 ligand (catalogue number G980B) and NanoBRET Nano-Glo substrate (catalogue number N157B) were from Promega Ltd.

*BCL6 TR-FRET assays*

Test compound was prepared as 11 point, 3 fold serial dilutions in DMSO from an upper concentration of 1 mM, DMSO only was used to generate the high control (0% inhibition) and a sufficient concentration of a control inhibitory compound used to generate the low control (100% inhibition). 10 μL of a mixture of BCL6-Avi-Bt (2.5 nM), D2-SMRT peptide (50 nM) and Europium Streptavidin (2 nM) in assay buffer (50 mM Hepes pH 7.4, 150 mM NaCl, 1 mM DTT, 1 mM Chaps) covered and incubated with compound at room temperature for 60 minutes. The plate was measured on an Envision plate reader. BCOR and NCOR assays were as above but substituting D2-SMRT peptide for 150nM AF647-BCOR and 25nM AF647-NCOR respectively.

*NanoBRET cell assay*

Assay day 1 (Transient Transfection): 8 x 10^6^ HEK293-WT cells per T75 flask (one per compound plate), were transfected with mix comprising 10 μg BCL6 DNA, 10 μg domain SMRT DNA, 1 mL Opti-MEM and 60 μL FuGene (added last), incubating at room temperature for 10 mins prior to addition to flask. The flask was incubated for 20-24 hours at 37 ˚C and 5% CO_2_. Assay day 2 (Cell plating): Cells were detached, spun and re-suspended at 2 x 10^5^ cells/mL, HaloTag NanoBRET 618 ligand at 1:1000 dilution was added and plated at 40 μL / well with test compounds prepared as above, a duplicate plate without HaloTag ligand was used as background control. The plates were incubated at 37 ˚C and 5% CO_2_ overnight (18-24 hours). Assay day 3 (Assay): 1:1000 NanoGlo substrate final was added to all wells, shaken for 30 seconds and luminescence then read on an Envision plate reader. The ‘no ligand’ control data were subtracted from the respective ‘plus ligand’ assay data before further analysis.

*TR-FRET and NanoBRET data Analysis*

Data were normalised between the robust mean of the high control and low control values using Activity Base (IDBS). Curve fitting was performed using the four-parameter logistic equation in ActivityBase: y=a-d/(1+(x/c)b)+d where ‘a’ is the minimum asymptote response, ‘b’ is the Hill slope, ‘c’ is the pIC50, and ‘d’ is the maximum asymptote response. The following values were used to initially define limits and/or constraints for each of the four parameters: Asymptote Minimum limits: - 20 – 20, Asymptote Maximum limits: 80 – 120, Slope limits: 0.5 – 2.5. Curves not resulting in a fit of the data as defined by these settings were intervened with manually by fixing the minimum, maximum and slope to individual or combinations of constraint/limit values.

*Flow Cytometry Of Spleen Cells*

Single cell suspensions were generated by passing spleens through a 40 µM cell strainer into medium (RPMI 1640 supplemented with 2% fetal calf serum, 1% penicillin / streptomycin, 1% L-glutamine). Following centrifugation (400 g, 5 minutes, 4 ˚C) and removal of supernatants, red blood cells were lysed by incubation in 1 mL lysis buffer for 1 minute at room temperature. Cells were resuspended in medium, counted on a Nucleocounter NC3000 (Chemometec, Lillerød, Denmark) and 1x10^6^ cells plated into 96-well polypropylene plates for staining. Cells were washed in 200 µL PBS and resuspended in 100 µL viability dye (diluted 1:1000 in PBS) for 15 minutes at room temperature. 100 µL cell staining buffer (CSB) (BioLegend, San Diego, CA, USA, #420201) was added to each well before centrifugation and removal of supernatants. Cell pellets were resuspended in 100 µL relevant cocktail of antibodies (see Tables S2 and S3) for surface staining and incubated for 30 minutes at 4 ˚C. Cells were washed in CSB and resuspended in 200 uL of FoxP3 fixation and permeabilisation buffer for 30 minutes in the dark at room temperature, washed and stained for intracellular markers for 30 minutes at room temperature. Cells were washed in Permwash and resuspended in CSB (250 µL) before acquisition on a Cytek Aurora spectral cytometer (Cytek, Fremont, CA, USA). Gating was carried out as shown (Figures S1 and S2) and data was analysed on FlowJo 10 (Becton Dickinson) and data was presented in Prism 7 (GraphPad, San Diego, CA, USA).

Statistical analysis of the data generated in this study was performed using R software. Data generated for serum IgG at day 5 were below the level of quantification and have therefore been excluded from the analysis. Absolute numbers of germinal centre B cells and T follicular helper cells were calculated from the frequency of the population of interest within the live cell gate. This frequency was used to calculate an absolute cell number per tissue, from the total live cell counts from whole homogenised spleen provided by the Nucleocounter NC3000 (Chemometec). To calculate the percent inhibition of GC and Tfh responses induced by TNP-KLH challenge, data was normalised against background GC and Tfh quantified in naive mice prior to performing percent inhibition calculations.

*Cytokine and chemokine ELISA*

To determine cytokine and chemokine levels plasma samples were analysed by electrochemiluminescence assay using a custom mouse multiplex kit (K15355K-4, Meso Scale Diagnostics (MSD), Rockville, MD, USA) to measure IFNγ, IL-6, IL-10, TNF, MCP-1, KC, IL-12, IL1b, IL-13 and IL17a according to the manufacturer’s protocol. Plates were read using an MSD Sector Imager 6000 and analysed with Discovery workbench software v4.0.11, cytokine data were back-fitted to the MSD standard curve.

*Gene expression analysis by semi-quantitative real time PCR*

Farage cells were plated at a density of 3000 cells/100 µl in 96-well plates, incubated overnight at 37 ˚C then treated with a 10-point, 1:3-fold serial dilution of compound at a top concentration of 20 µM for GSK137 or 10 µM BI3802. Cells were returned to the incubator for 3 days prior to harvesting for RNA isolation. RNA was isolated using the TurboCapture 96 Kit (Qiagen, Hilden, Germany; # 72251), cDNA generated using the High Capacity cDNA Reverse Transcription kit (ABI, Foster City, CA, USA; #4374966) both following the manufacturer’s instructions. qPCR was performed on a ViiA7 real-time PCR machine (Life Technologies, Carlsbad, CA, USA) using the Fast TaqMan ΔΔCt method. Quadruplicate readings were measured for each biological replicate sample. Individual TaqMan assays are listed (Table S4). Expression data were analyzed using the Comparative Ct^(2–ΔΔCt)^ method (42) The Ct values of the treated samples are compared to vehicle treated samples and normalized to GAPDH. Changes are presented as an average RQ (termed “relative expression”). RQ values between 0 and 1 correspond to a downregulation of gene expression, RQ values greater than 1 correspond to upregulation.

*General synthetic route to GSK137*

Synthesis of GSK137 is shown in Figure S3.

*Step 1: Pyrazolo[1,5-a]pyrimidine-5,7-diol 2*

A solution of 1 (250 g, 3.01 mol) and diethyl malonate (482 mL, 3.16 mol) in MeOH (2 L) was added drop-wise to a solution of 25% wt NaOMe (688 mL, 3.01 mol) in MeOH at 20 °C. The reaction was then heated to 80 °C under a N_2_ atmosphere for 16 hours. The reaction mixture was cooled to RT and the resulting solids were filtered. The solid was suspended in water (2 L), the pH was adjusted to 2-3 with 3N HCl (300 mL) and the mixture was stirred for 30 min. The solids were filtered, and the filtrate was treated with NaCl (~250 g) and stirred, resulting in further solid formation. This new solid was filtered through the same funnel to combine with the initially formed solid. The compound was dried under reduced pressure and then twice evaporated from toluene (2 L) and dried to give the title compound 2 as an off-white solid (453 g, 2.88 mol, 96%). 1H-NMR (400 MHz, DMSO-d6): δ = 11.44 (1H, br.s), 7.62 (1H, br.s), 4.53 (1H, br.s) ppm. LCMS (2 min, ES+, formic): Rt = 0.18 min, [M+H]+ = 152.

*Step 2: 5,7-Dichloropyrazolo[1,5-a]pyrimidine 3*

Under N2 2 (453 g, 3 mmol) was added portion-wise to stirred POCl3 (2.79 L, 30 mol) over 40 minutes. N,N-dimethylaniline (380 mL, 3.0 mol) was added drop-wise after which the reaction was heated under reflux for 16 h. The reaction mixture was cooled to RT and the volatiles were removed under reduced pressure. The crude material was dissolved in MeCN (3 L), quenched with saturated Na2CO3 (16 L) and extracted with EtOAc (3x5 L). The combined organic layers were washed with 1.5N HCl (3 L) and the separated organic layer was dried over anhydrous Na2SO4 (~100 g) and filtered. The filtrate was concentrated under reduced pressure to give the title compound 3 as a yellow solid (320 g, 1.7 mol, 57%). 1H-NMR (400 MHz, DMSO-d6): δ = 8.40 (1H, d, J=2.5 Hz), 7.68 (1H, s), 6.89 (1H, d, J=2.5 Hz) ppm. LCMS (2 min, ES+, formic): Rt = 0.74 min, [M+H]+ = 188.

*Step 3: 5,7-Dichloro-3-fluoropyrazolo[1,5-a]pyrimidine 4*

A suspension of 3 (140 g, 745 mmol) and selectfluor (528 g, 1.5 mol) in water (2.2 L) was heated at 90 °C for 16 hours. The reaction mixture was cooled to RT and filtered, washing through with water (500 mL). The filtrate was extracted with EtOAc (2 L) and the organic layer was dried over anhydrous Na_2_SO_4_ (100 g), filtered and concentrated under reduced pressure to give crude compound as a light brown solid (165 g). The crude compound was purified using flash column chromatography on silica gel eluting with 2% EtOAc in petroleum ether. Desired fractions were combined and concentrated to give the title compound 4 as a solid (40.1 g, 26%). Mixed fractions were also combined and concentrated to give a second (less pure) batch (20.6 g). This second batch was re-purified under the same column conditions to give pure product 4 (8.6 g, 6%). 1H-NMR (400 MHz, CDCl3): δ = 8.17 (1H, d, J=3.5 Hz), 7.01 (1H, s) ppm. LCMS (2 min, ES+, formic): Rt = 0.74 min, [M+H]+ = 206.

*Step 4: 5-Chloro-3-fluoro-7-(2-methylpyridin-3-yl)pyrazolo[1,5-a]pyrimidine 5*

Compound 4 (25 g, 121 mmol), (2-methyl-3-(4,4,5,5-tetramethyl-1,3,2-dioxaborolan-2-yl)pyridine (23.9 g, 109 mmol) and K2CO3 (33.5 g, 243 mmol) were combined in 1,4-dioxane (375 mL) and water (125 mL). After degassing with N2 for 15 min followed by evacuation and N_2_ back-fill, PdCl2(dppf).CH_2_Cl_2_ (8.92 g, 10.9 mmol) was added. The mixture was heated at 100 °C for 2.5 h. The reaction mixture was cooled to RT and filtered through celite, washing through with EtOAc (500 mL). The filtrate was concentrated and the resulting crude material was taken up in EtOAc (800 mL) and washed with water (200 mL) and brine solution. The separated organic layer was dried over anhydrous Na_2_SO_4_ (~15 g) and filtered. The filtrate was concentrated under reduced pressure to give the crude compound as light brown gummy solid (40.6 g). The crude was triturated with MeCN (30 mL) and the resulting solid was filtered and dried under reduced pressure to give the desired compound 5 as a light brown solid (15.9 g, 59.9 mmol, 49%). The filtrate was concentrated to give crude compound (24.1 g) as a brown liquid. This was purified by flash column chromatography on silica gel eluting with a graduating solvent system of 0-100% EtOAc/petroleum ether. The desired fractions were combined and concentrated to give the title compound 5 as a light brown solid (7.16 g, 23.8 mmol, 20%). 1H-NMR (400 MHz, DMSO-d6): δ = 8.69 (1H, dd, J=5, 1.5 Hz), 8.43 (1H, d, J=3.0 Hz), 7.94 (1H, dd, J=7.5, 1.5 Hz), 7.45 (1H, dd, J=7.5, 5.0 Hz), 7.39 (1H, s), 3.95 (3H, s) ppm. LCMS (2 min, ES+, formic): Rt = 0.75 min, [M+H]+ = 263.

*Step 5: N-(tert-Butyl)-5-(3-fluoro-7-(2-methylpyridin-3-yl)pyrazolo[1,5-a]pyrimidin-5-yl)quinolin-2-amine 6*

Compound 5 (15.0 g, 57.1 mmol), compound 11 (20.49 g, 62.8 mmol) and K2CO3 (15.8 g, 114 mmol) were combined in 1,4-dioxane (225 mL) and water (75 mL). After degassing with N_2_ for 20 min followed by evacuation and back-fill with N2, PdCl2(dppf).CH_2_Cl_2_ (4.20 g, 5.14 mmol) was added. The mixture was heated at 100 °C for 2.5 h. The reaction mixture was cooled to RT and filtered through celite, washing through with EtOAc (500 mL). The filtrate was removed under reduced pressure to give the crude compound. This was taken up in EtOAc (600 mL) and washed with water (200 mL) and brine solution. The separated organic layer was dried over anhydrous Na2SO4 (~20 g) and filtered. The filtrate was removed under reduced pressure to give the crude compound as a dark brown foamy solid (30.6 g). Purification was undertaken using flash column chromatography on silica gel. The product was eluted using a graduating solvent system of 0-100% EtOAc/petroleum ether. The desired fractions were combined and concentrated to give the title compound 6 as a light yellow foamy solid (11.7 g, 26.3 mmol, 46%). 1H-NMR (400 MHz, DMSO-d6): δ = 8.68 (1H, dd, 5.0, 1.5 Hz), 8.41 (1H, d, J=3.5 Hz), 8.32 (1H, d, J=9.5 Hz), 8.04 (1H, dd, J=8.0, 2.0 Hz), 7.67-7.44 (4H, m), 7.43 (1H, s), 6.85 (1H, d, J=9.0 Hz), 6.81 (1H, s), 2.38 (3H, s), 1.51 (9H, s) ppm. LCMS (2 min, ES+, formic): Rt = 0.65 min, [M+H]+ = 427.

*Step 6: cis-N-(tert-Butyl)-5-(3-fluoro-7-(2-methylpyridin-3-yl)-4,5,6,7-tetrahydropyrazolo[1,5-a]pyrimidin-5-yl)quinolin-2-amine 7*

A solution of 6 (11.5 g, 27.0 mmol) in MeOH (120 mL) was stirred under N_2_. To this was added NaBH4 (5.10 g, 135 mmol) portion-wise at RT. The reaction mixture was stirred for 3.5 hours after which, further NaBH4 (5 eq) was added portion-wise at RT every 30-40 minutes (x4). The reaction was quenched by the addition of crushed ice resulting in solid formation. The solids were filtered and washed with water (100 mL), dried under reduced pressure to give the title compound 7 as a yellow solid (10.6 g, 23.6 mmol, 88%). 1H-NMR (400 MHz, DMSO-d6): δ = 8.35 (1H, dd, 5.0, 1.5 Hz), 8.29 (1H, d, J=9.0 Hz), 7.47-7.42 (4H, m), 7.27 (1H, d, J=4.0 Hz), 7.18 (1H, dd, J=8.0, 5.0 Hz), 6.84 (1H, d, J=9 Hz), 6.68 (1H, s), 6.59 (1H, s), 5.77 (1H, dd, J=11.0, 5.0 Hz), 5.26 (1H, d, J=11.0 Hz), 2.33 (3H, s), 1.48 (9H, s) ppm. LCMS (2 min, ES+, formic): Rt = 0.42 min, [M+H]+ = 431.

*Step 7: 5-((5S,7R)-3-Fluoro-7-(2-methylpyridin-3-yl)-4,5,6,7-tetrahydropyrazolo[1,5-a]pyrimidin-5-yl)quinolin-2-amine GSK137*

A solution of 7 (51.5 mmol) in TFA (240 mL, 3.1 mol) was stirred under N2 at 70 °C for 16 h. The reaction was cooled to RT and concentrated in vacuo. The residue was washed with Et2O (2x200 mL) then the resulting solids were basified to pH~9 with 10% Na2CO3 solution and extracted with EtOAc (3x400 mL). The combined organic layers were washed with water (200 mL) and brine solution (100 mL) and then dried over Na2SO4 (10.5 g), filtered and concentrated to give the crude product as an off-white solid. Purification was undertaken via chiral SFC on a PIC 400 instrument using a LUX C3 (250*50 mm, 5 µm) column. The product was eluted with CO_2_/0.5% iPrNH2 in MeOH (60:40) at a flow rate of 180 g/min (back pressure = 100 bar). The first eluting fraction (desired enantiomer) was concentrated under reduced pressure. The resulting residue was triturated Et2O (x3) and the solid was filtered and dried in vacuo. The material was dissolved in MeOH (80 mL) and the solution was added to water (800 mL) resulting in the formation of a solid. The solid was filtered and dried in vacuo to give the title compound as an off-white solid (18%). 1H-NMR (400 MHz, DMSO-d6): 8.40 (1H, d, J=9.0 Hz), 8.35 (2H, dd, J=5.0, 1.5 Hz), 7.51-7.39 (4H, m), 7.27 (1H, d, J=4.5 Hz, 1H), 7.18 (1H, q, J=5.0 Hz), 6.82 (1H, d, J=9.0 Hz), 6.60 (1H, s), 6.43 (2H, s), 5.76-5.77 (1H, m), 5.28 (1H, d, J=11.0 Hz), 2.50-2.40 (4H, m), 2.18-2.00 (1H, q, J=11.0 Hz). LCMS (2 min, ES+, formic): Rt = 1.94 min, [M+H]+ = 375. Chiral SFC (Lux C3, CO2/40% 0.5% iPrNH2 in MeOH, 3 mL/min): Isomer 1(GSK137) Rt = 1.88 min (99.6%), Isomer 2 Rt = 2.69 min (0.4%).

*Step A: 5-Bromoquinoline 1-oxide 9*

A solution of 8 (25.0 g, 120 mmol) in CH_2_Cl_2_ (500 mL) was stirred under N_2_ and cooled to 0 °C. m-CPBA (57.6 g, 240 mmol) was added and the reaction mixture was stirred at RT for 16 h. The reaction was quenched by the addition of 10% NaOH solution, adjusting to pH~8, and extracted with CH2Cl2 (3x200 mL). The combined organic layers were washed with water (2x100 mL), saturated brine solution (100 mL) and separated. The organic layer was dried over anhydrous Na2SO4 (5.0 g), filtered and concentrated in vacuo to give the title compound 9 as an off-white solid (27 g, 120 mmol, quant.). 1H-NMR (400 MHz, DMSO-d6): δ = 8.69 (1H, dd, J=6.0, 1.0 Hz), 8.58 (1H, d, J=9.0 Hz), 8.11 (1H, dd, J=7.5, 1.0 Hz), 8.02 (1H, d, J=9.0 Hz), 7.74 (dd, J=7.5, 7.5 Hz), 7.62 (1H, dd, J=9.0, 6.0 Hz) ppm. LCMS (2 min, ES+, formic): Rt = 0.71 min, [M+H]+ = 224, 226.

*Step B: 5-Bromo-N-(tert-butyl)quinolin-2-amine 10*

A stirred solution of 9 (27.0 g, 121 mmol) in CH2Cl2 (500 mL) stirred was cooled to 0 °C under N2. p-Ts2O (79 g, 241 mmol) was added portion-wise followed by 2-methylpropan-2-amine (63.3 mL, 603 mmol). The reaction mixture was stirred at RT for 16 h. The reaction was quenched with water (100 mL) and extracted with EtOAc (2x300 mL) and separated. The combined organic layers were washed with water (2x100 mL), brine solution (100 mL) and separated. The organic layer was dried over anhydrous Na2SO4 (5.6 g), filtered and concentrated to give the crude product (46.0 g). Purification was achieved via column chromatography on silica gel. The product was eluted using a graduating solvent system of 0-5% EtOAc in petroleum ether. The required fraction were combined and concentrated in to give the title compound 10 as a brown solid (20.2 g, 67.2 mmol, 56%). 1H-NMR (400 MHz, DMSO-d6): δ = 7.94 (1H, dd, J=9.0, 0.5 Hz), 7.49 (1H, dt, J=8.0, 1.0 Hz), 7.42 (1H, dd, J=7.5, 1.0 Hz), 7.36 (1H, dd, J=8.0 Hz), 6.97 (1H, s), 6.89 (1H, d, J=9.0 Hz) 1.48 (9H, s) ppm. LCMS (2 min, ES+, formic): Rt = 0.73 min, [M+H]+ = 279, 281.

*Step C: N-(tert-Butyl)-5-(4,4,5,5-tetramethyl-1,3,2-dioxaborolan-2-yl)quinolin-2-amine 11*

Compound 10 (18.0 g, 64.5 mmol), B2pin2 (19.7 g, 77 mmol) and KOAc (19 g, 193 mmol) were combined in 1,4-dioxane (400 mL). After degassing with N2 for 15 min followed by evacuation and back-filling with N2, PdCl2(dppf).CH2Cl2 (5.27 g, 6.45 mmol) was added. The mixture was heated at 100 °C for 3 hours and cooled to RT. The mixture was filtered through celite and the filtrate was concentrated in vacuo to give the crude compound (35.1 g). Purification was achieved via flash column chromatography on silica gel. The product was eluted using a graduating solvent system of 0-100% EtOAc/petroleum ether. The desired column fractions were combined and concentrated under reduced pressure to give the title compound 11 as a light yellow liquid (20.6 g, 46.3 mmol, 72%). 1H-NMR (400 MHz, DMSO-d6): δ = 8.43 (1H, d, J=9.0 Hz), 7.61-7.56 (2H, m), 7.44 (1H, m), 6.79 (1H, d, J=9.0 Hz), 6.64 (1H, s) 1.48 (9H, s), 1.35 (12H, s) ppm. LCMS (2 min, ES+, formic): Rt = 0.80 min, [M+H]+ = 327.

**Table S1**

Data collection and refinement statistics (Molecular replacement).

| **Data collection** | |
| --- | --- |
| **Space Group** | P3_2_21 |
| **Cell Dimensions** |  |
| **a,b,c (Å)** | 49.251, 49.251, 124.409 |
| **α, β, γ (°)** | 90.00,90.00, 90.00 |
| **Diffraction limits (Å)** | 2.40, 2.40, 1.85 |
| **Resolution (Å)** | 2.04 (2.25-2.04) |
| **R_merge_*^b^*** | 0.167 (2.195) |
| **R_meas_*^c^*** | 0.178 (2.314) |
| **Average *I/σI*** | 11.0 (1.5) |
| **Completeness (ellipsoidal) (%)** | 82.8 (42.9) |
| **Redundancy** | 10.4 (10.0) |
| **No. Reflections** | 79104 (3791) |
| **No. Unique Reflections** | 7572 (379) |
| **Refinement** | |
| **Resolution (Å)** | 20.00-2.04 |
| **R_work_/R_free_** | 0.180/0.241 |
| **No. Reflections** | 7062 |
| **No. atoms** |  |
| **Protein (A)** | 1008 |
| **Cmpd (L)** | 28 |
| **GOL (G)** | 6 |
| **DMS (D)** | 4 |
| **HOH (W)** | 94 |
| **B-factors [Å^2^]** |  |
| **Protein (A)** | 50.4 |
| **Cmpd (L)** | 42.6 |
| **GOL (G)** | 89.3 |
| **DMS (D)** | 90.8 |
| **HOH (W)** | 50.9 |
| **R.M.S. deviations** | |
| **Bond lengths (Å)** | 0.006 |
| **Bond angles (°)** | 1.402 |

*^a^* Data for the highest resolution shell are given in parentheses.

*^b^* R_merge_ = Σ|*I_j_* - <*I_j_*>|/Σ<*I_j_*>.

*^c^* R_meas_ = Σn_j_(n_j_ -1) Σ|*I_j_* - <*I_j_*>|/Σ<*I_j_*>.

**Table S2**

Antibody panels for flow cytometry.

**Table S3**

Reagents and antibodies for flow cytometry.


**Table S4**

TaqMan assays used in the study. All assays were purchased from Life Technologies.

**Figure S1**

Sequential gating strategy for identification of germinal center B cells. Leukocytes were selected by forward and side scatter, doublets and dead cells excluded, CD11b^+^ NK1.1^+^ and CD11c^+^ cells were excluded via the dump channel, B cells were selected through CD3e-, CD19+ and germinal centre B cell subset identified through the expression of Bcl6 and GL7. As confirmation that the Bcl6^+^ GL7^+^ population contained germinal centre B cells, expression of additional germinal centre B cell marker FAS and PNA were verified (bottom right plot, red events show FAS and PNA expression of Bcl6^+^ GL7^+^ B cells, blue events shows FAS and PNA expression of remaining B cells).

**
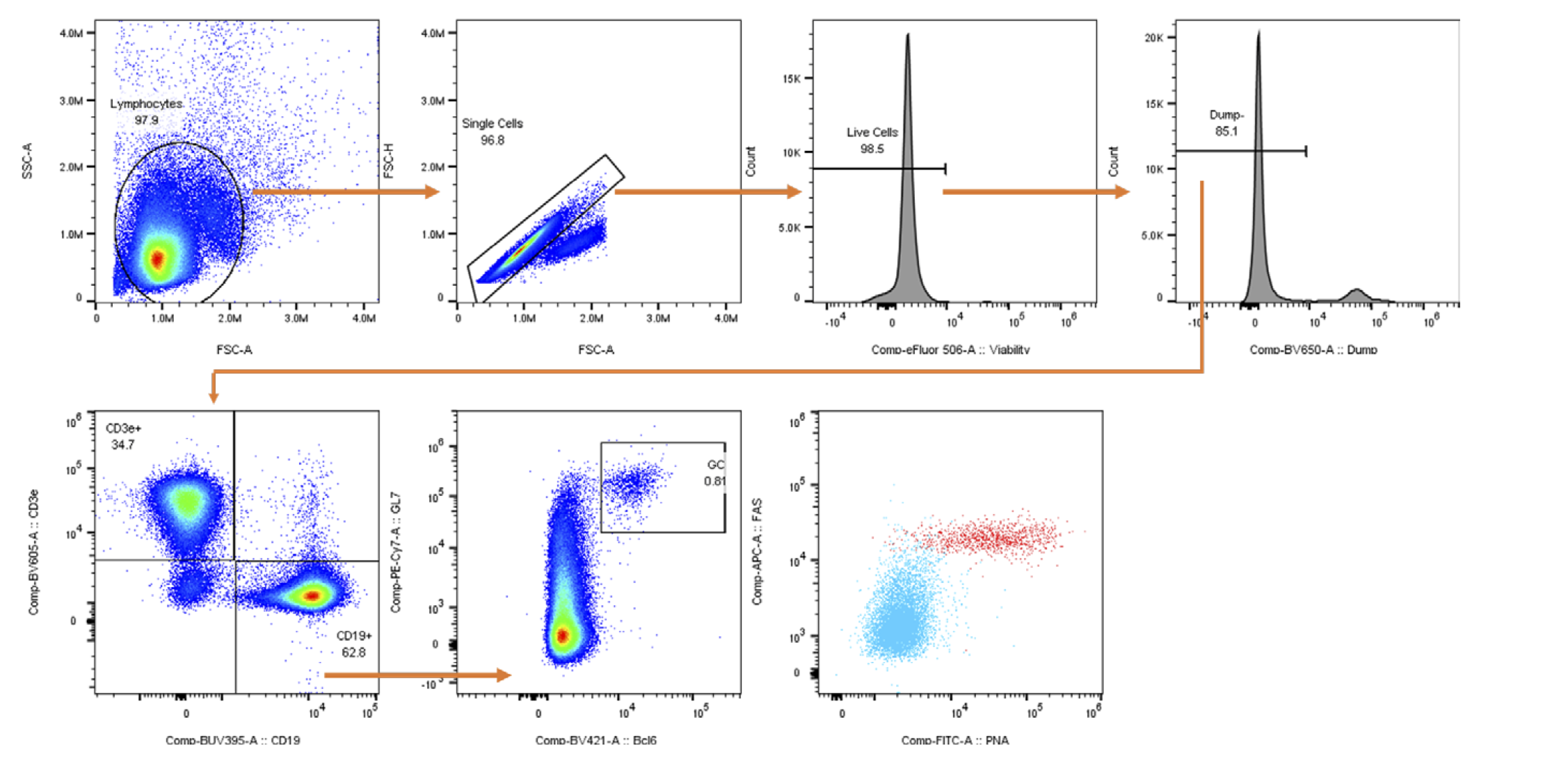
**

**Figure S2**

Sequential gating strategy for identification of Tfh cells. Leukocytes were selected by forward and side scatter, doublets and dead cells excluded, CD11b^+^ NK1.1^+^ and CD11c^+^ cells were excluded via the dump channel, T helper cells were selected through CD19^-^, CD3e^+^, CD4^+^ and TfH subset identified through the expression of CXCR5 and PD-1.

**
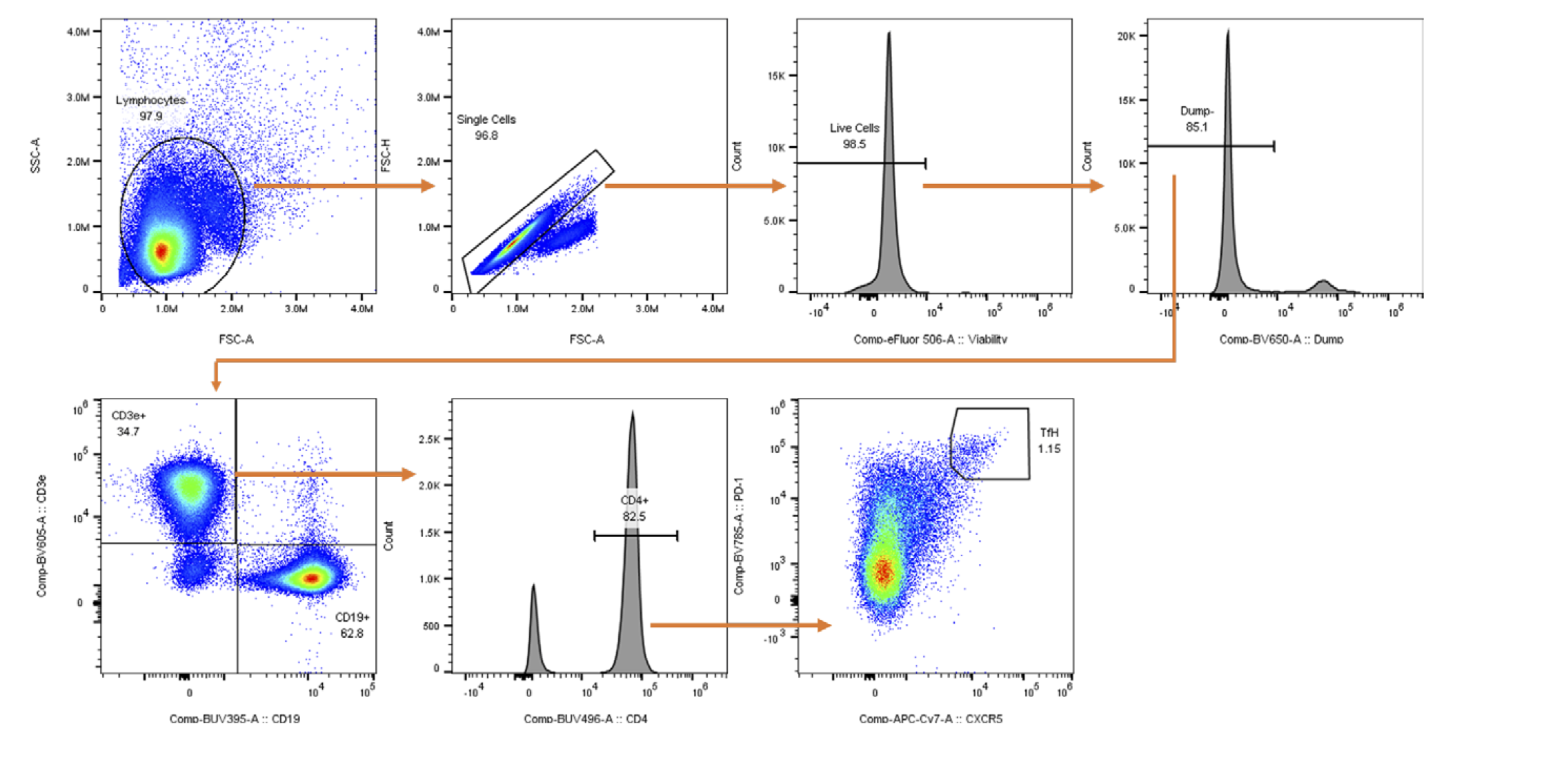
**

**Figure S3**

General synthetic routes to GSK137.

**
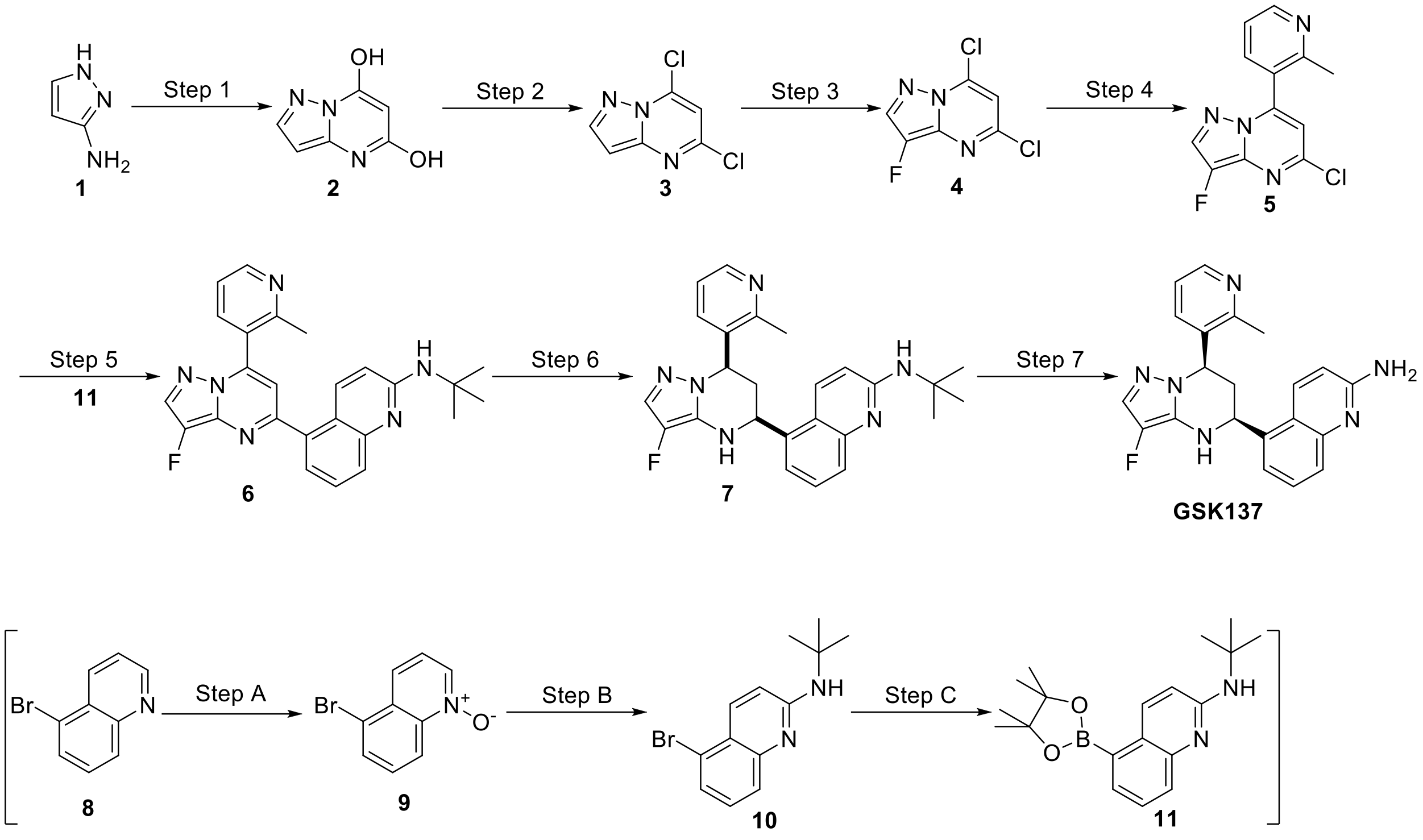
**

**Figure S4**

Western blot from Figure 1E shown complete for transparency purposes.


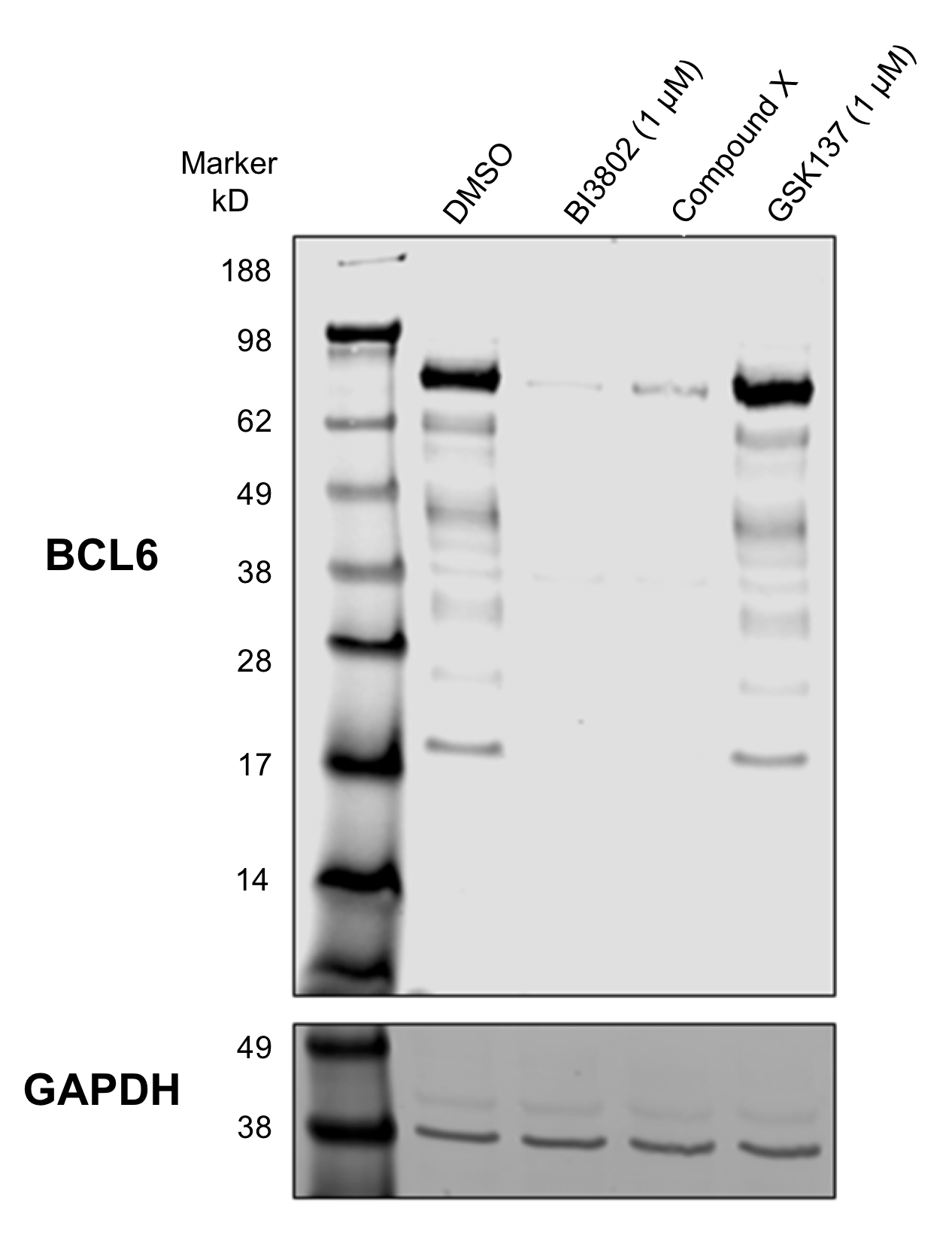


**Figure S5**


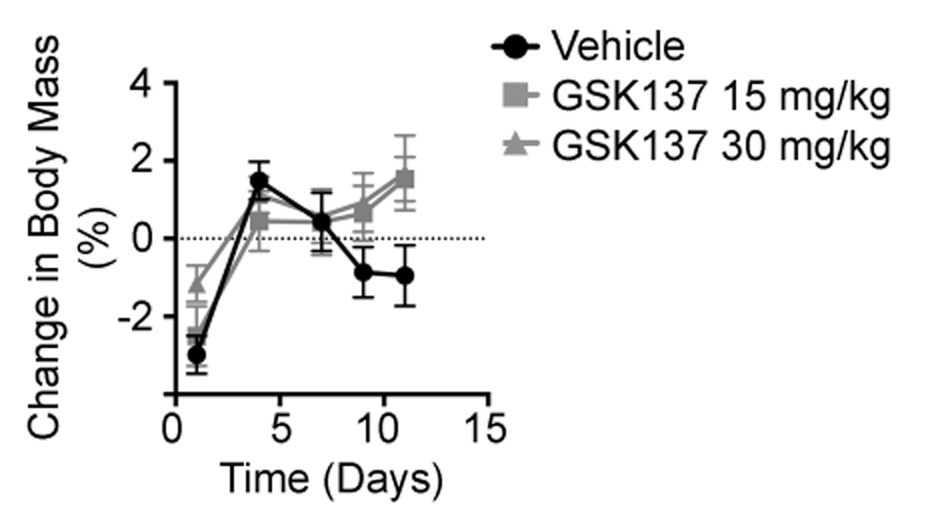
Body weight change expressed as percentage change relative to weight on day -1. Data are group mean ± SEM (vehicle treated animals n=14, GSK 15 mg/kg n=13, GSK137 30 mg/kg n=14). Animals were weighed on day -1 and then on days 1, 4, 7, 9 and 11 post-immunisation.

**Figure S6**

(A) Absolute number of plasmablasts and plasma cells (CD11b^-^CD11c^-^CD161^-^CD3e^-^CD19^+/int^CD138^+^) in vehicle and GSK137 treated groups. There is no statistically significant difference between the groups. (B) Plasmablasts and plasma cells expressed as a percentage of total viable cells in each of the experimental groups. There are again no statistically significant reductions in the fraction of plasmablasts with GSK137 (15 mg/kg) or GSK137 (30 mg/kg). Horizontal bars indicate mean values.


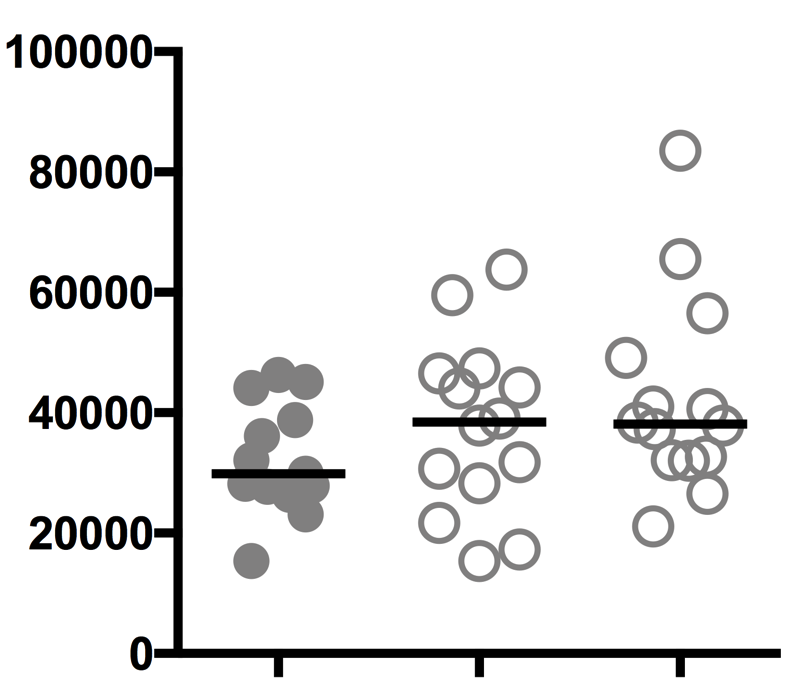

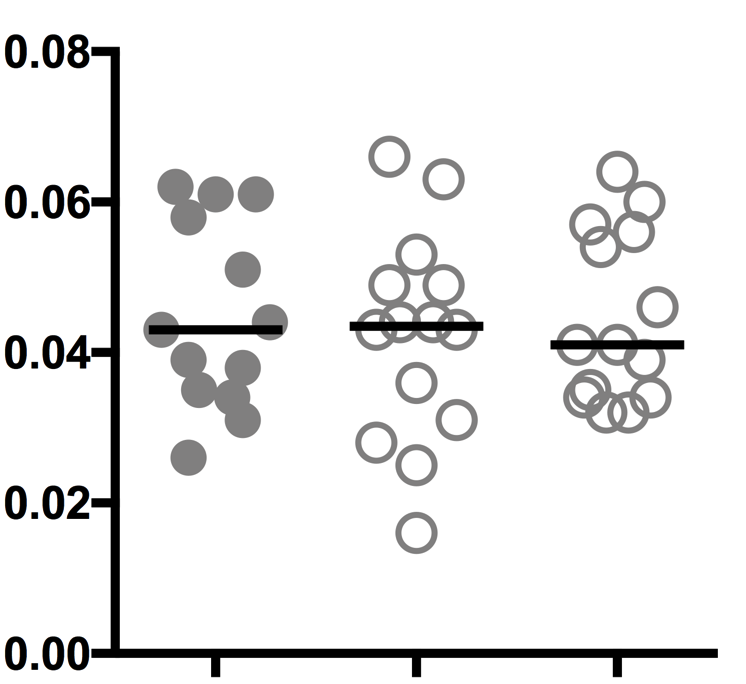


Control

GSK137 (15 mg/kg)

GSK137 (30 mg/kg)

Number of CD19^+/int^CD138^+^ cells

Control

GSK137 (15 mg/kg)

GSK137 (30 mg/kg)

CD19^+/int^CD138^+^ cells/Total

number of viable cells (%)

**A**

**B**

**Figure S7**

Scatter diagram showing numbers of germinal centers per follicle in immunized and vehicle treated animals versus immunized and GSK137 (15 mg/kg) treated animals. Each data point is the mean of two technical replicates for each control animal or animal treated with GSK137. Mean is indicated by the horizontal line. There is a significant difference between groups (Mann-Whitney U-test, P=0.04). Statistical differences are shown *p<0.05.


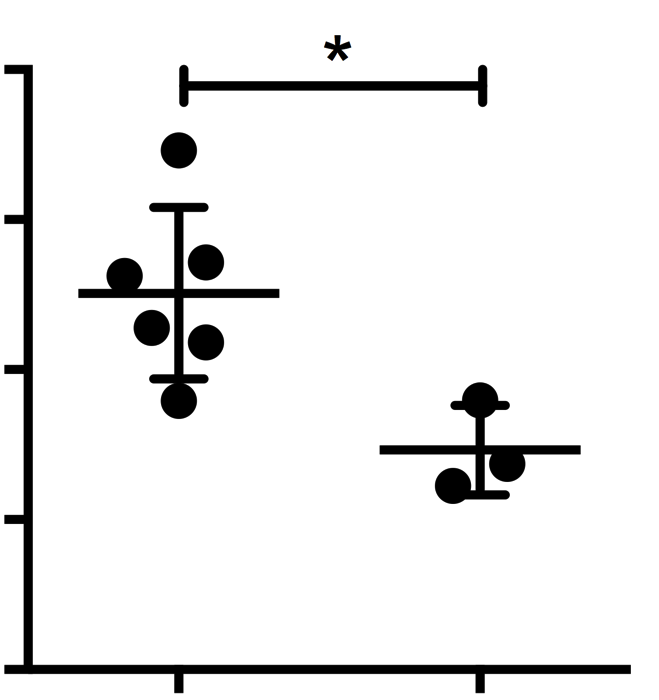


0.2

0.4

0.6

0.8

Numbers of

GC/Follicle

Control

GSK137
